# Variability in Macro- and Micronutrients of 15 Rarely Researched Microalgae

**DOI:** 10.3390/md21060355

**Published:** 2023-06-09

**Authors:** Fabian Sandgruber, Annekathrin Gielsdorf, Benjamin Schenz, Sandra Marie Müller, Tanja Schwerdtle, Stefan Lorkowski, Carola Griehl, Christine Dawczynski

**Affiliations:** 1 Junior Research Group Nutritional Concepts, Institute of Nutritional Sciences, Friedrich Schiller University, 07743 Jena, Germany; fabianalexander.sandgruber@uni-jena.de (F.S.); benjamin.schenz@uni-jena.de (B.S.); 2 Competence Cluster for Nutritional and Cardiovascular Health (nutriCARD) Halle-Jena-Leipzig, Dornburger Str. 25, 07743 Jena, Germany; stefan.lorkowski@uni-jena.de; 3 Competence Center Algal Biotechnology, Anhalt University of Applied Sciences, 06406 Bernburg, Germany; 4 Department of Food Chemistry, Institute of Nutritional Science, University of Potsdam, 14469 Potsdam, Germany; sandramarie.mueller@cvua-westfalen.de (S.M.M.); tanja.schwerdtle@bfr.bund.de (T.S.); 5 German Federal Institute for Risk Assessment (BfR), 10589 Berlin, Germany; 6 Institute of Nutritional Sciences, Friedrich Schiller University, 07743 Jena, Germany

**Keywords:** microalgae, protein, minerals, trace elements, fatty acids, omega-3 fatty acids, nutrition, health

## Abstract

Microalgae have enormous potential for human nutrition, yet the European Commission has authorized the consumption of only eleven species. Strains of fifteen rarely researched microalgae from two kingdoms were screened regarding their nutritional profile and value for human health in two cultivation phases. Contents of protein, fiber, lipids, fatty acids, minerals, trace elements and heavy metals were determined. In the growth phase, microalgae accumulated more arginine, histidine, ornithine, pure and crude protein, Mg, Mn, Fe and Zn and less Ni, Mo and I_2_ compared to the stationary phase. Higher contents of total fat, C14:0, C14:1_n5_, C16:1_n7_, C20:4_n6_, C20:5_n3_ and also As were observed in microalgae from the chromista kingdom in comparison to microalgae from the plantae kingdom (*p* < 0.05). Conversely, the latter had higher contents of C20:0, C20:1_n9_ and C18:3_n3_ as well as Ca and Pb (*p* < 0.05). More precisely, *Chrysotila carterae* appeared to have great potential for human nutrition because of its high nutrient contents such as fibers, carotenoids, C20:6_n3_, Mg, Ca, Mn, Fe, Se, Zn, Ni, Mo and I_2_. In summary, microalgae may contribute to a large variety of nutrients, yet the contents differ between kingdoms, cultivation phases and also species.

## 1. Introduction

Microalgae are rich in various essential nutrients such as macronutrients (proteins and amino acids, fats and omega-3 polyunsaturated fatty acids (n3-PUFA), carbohydrates and fibers) as well as micronutrients (vitamins, pigments, minerals and trace elements) [1]. Although microalgae have enormous potential for human nutrition, the European Commission has authorized the consumption of only eleven microalgae species [2,3]. So far, partial research and nomenclature has been carried out on 30,000 microalgae species of an estimated variety of 50,000 species [4]. Previous studies indicate differences in nutrient profile depending on the cultivation and growth period [5,6,7]. The cultivation of microalgae can be divided into five phases: induction phase, growth phase, phase of declining relative growth, stationary phase and death phase [8]. The first phase is characterized by a small increase in cell density because of the adaptation to changing culture condition from the former to new cultivation conditions. In the exponential or growth phase, the number of cells increases close to exponentially due to cell division. When nutrients, carbon dioxide and light supply decrease, the cell division rate drops, and the microalgae reach the third phase. Afterwards, the stationary phase begins, where the cell density is close to a constant level because of the balance between the growth rate and factors limiting growth. In the last phase, the death phase, nutrients are exhausted, which inhibits further growth, causing the microalgae density to decrease, and the culture collapses [8]. Whereas parameters such as different growth media composition, temperature and light quality or quantity are commonly used to customize the nutrient profile of microalgae, differences in the nutrient profile between the cultivation phases have been studied only to some extent. For instance, various studies indicate a higher content of polyunsaturated fatty acids (PUFAs) and phytochemicals such as β-carotene in the growth phase rather than in the stationary [9,10,11,12].

The aim of the present study was to evaluate the nutrient profile of 15 rarely studied microalgae species from the kingdoms plantae (*Autumnella lusatica*, *Botryococcus braunii*, *Chlorococcum novae-anglia*, *Klebsormidium* sp., *Myrmecia bisecta*, *Spongiochloris minor*, *Stichococcus* sp., *Tetradesmus obliquus*, *Tetraselmis suecica*) and chromista (*Chrysotila carterae*, *Eustigmatos* sp., *Microchloropsis salina*, *Nannochloropsis limnetica*, *Nitzschia palea*, *Phaeodactylum tricornutum)*, which showed valuable amounts of different nutrients in pre-screening tests. The analyzed nutrient profile of all microalgae has been used to rate their potential benefit for human nutrition. Furthermore, differences in the nutrient profile of four microalgae species during cultivation in the growth phase and stationary phase have been analyzed, compared and categorized using their nutritional value. The microalgae species were selected based on literature searches according to the criteria of presumed nutrient profile, cultivability and food safety, whereby the latter was assessed based on relatedness to approved species and on toxin formation study results.

## 2. Results

### 2.1. Amino Acid Analysis

The amino acid profiles did not differ significantly between the microalgae biomass of the two kingdoms. The specific N-factors (nitrogen-to-protein conversion factor) for each microalgae were neither different between kingdoms nor between cultivation phases (Table 1).

Arginine, histidine and ornithine contents were higher in the growth phase compared to the stationary phase (*p* < 0.05; Table 1). In the growth phase, arginine ranged from 1.3 (*S. minor*) to 3.0 g/100 g d.w. (*T. obliquus*) and in the stationary phase from 0.6 (*M. salina*) to 3.2 g/100 g d.w. (*S. minor*). Histidine concentrations were from 0.4 (*S. minor*) to 0.8 g/100 g d.w. (*M. salina*) in the growth phase, while in the stationary phase, they ranged from 0.3 (*M. salina*) to 0.4 g/100 g d.w. (*S. minor*). Ornithine concentrations in the growth phase were between 0.03 (*S. minor)* and 0.12 g/100 g d.w. (*T. obliquus*), while in the stationary phase, they were between 0.02 (*M. salina*) and 0.06 g/100 g d.w. (*S. minor*).

The nonessential amino acid (NEA) concentration in chromista varied between 4.7 (*C. novae-angliae*) and 12.7 g/100 g d.w. (*N. palea*) and in plantae from 5.0 (*T. suecica*) to 14.4 g/100 g d.w. (*S. minor*; Table 1). In the growth phase, the range was between 9.7 (*S. minor*) and 17.1 g/100 g d.w. (*C. novae-angliae*), while in the stationary phase, it was between 4.7 (*C. novae-angliae*) and 14.4 g/100 g d.w. (*S. minor*). Between kingdoms, the semi-essential amino acid (SEA) concentration varied from 1.0 (*P. tricornutum*) to 1.7 g/100 g (*C. carterae*) in chromista and 0.9 (*Stichococcus* sp.) to 3.6 g/100 g d.w. (*S. minor*) in plantae (Table 1). SEA concentrations in microalgae in the growth phase were from 1.6 (*S. minor*) to 2.8 g/100 g d.w. (*M. salina*) and in the stationary phase from 0.9 (*C. novae-angliae*) to 3.6 g/100 g d.w. (*S. minor*). The range of essential amino acid (EAA) concentrations was from 4.1 (*M. salina*) to 8.9 g/100 g (*C. carterae*) in chromista and 4.9 (*Stichococcus* sp.) to 11.1 g/100 g d.w. (*S. minor*) in plantae (Table 1). EAA ranged from 7.4 (*T. obliquus*) to 13.5 g/100 g (*C. novae-angliae)* in the growth phase and 4.1 (*C. novae-angliae)* to 11.1 g/100 g d.w. (*S. minor*) in the stationary phase. 

N-factors ranged from 4.38 (*M. salina*) to 5.47 (*P. tricornutum*) in chromista and 4.42 (*C. novae-angliae*) to 5.61 (*S. minor*) in plantae. In the growth phase, the N-factors ranged from 3.93 (*M. salina* and *C. novae-angliae*) to 4.77 (*S. minor*). 

### 2.2. Nitrogenous Compounds

The analysis of nitrogenous compounds in microalgae did not reveal any significant differences in total fiber, nitrogen or non-protein nitrogen (NPN) between both kingdoms or in different cultivation phases (*p* > 0.05; Table 2). 

In chromista, the crude protein content (use of specific N-factor) was between 10.7 (*M. salina*) and 24.0 g/100 g d.w. (*N. palea*), while in plantae, it was between 10.2 (*T. suecica*) and 30.4 g/100 g d.w. (*A. lusatica*). Higher contents of crude protein were detected in biomass in the growth phase, ranging from 20.5 (*T. obliquus*) to 32.0 g/100 g d.w. (*C. novae-angliae*), compared to the stationary phase, ranging from 10.7 (*M. salina*) to 22.7 g/100 g d.w. (*S. minor*; *p* < 0.05; Table 2).

The pure protein content in chromista ranged from 10.0 (*M. salina*) to 20.3 g/100 g d.w. (*N. palea*) and in plantae from 7.7 (*T. suecica*) to 23.1 g/100 g d.w. (*Klebsormidium* sp.). It was higher in the growth phase compared to the stationary phase, ranging from 16.0 (*T. obliquus*) to 28.2 g/100 g d.w. (*M. salina*) and from 10.0 (*M. salina*) to 21.0 g/100 g d.w. (*S. minor*), respectively (*p* < 0.05; Table 2). 

The total fiber content in chromista varied from 14.3 (*N. limnetica*) to 41.2 g/100 g (*C. carterae*) and in plantae from 13.7 (*M. bisecta*) to 40.5 g/100 g (*S. minor*; Table 2). In the growth phase, the range was from 23.8 (*M. salina*) to 40.4 g/100 g d.w. (*S. minor*) and in the stationary phase from 21.0 (*M. salina*) to 40.5 g/100 g d.w. (*S. minor*).

**Table 1:** Amino acid profiles and ammonium contents of 15 microalgae from different kingdoms and cultivation phases in g/100 g d.w. with their calculated N-factor.

Species	*Chrysotila carterae*	*Eustigmatos* sp.	*Microchloropsis salina*	*Nannochloropsis limnetica*	*Nitzschia palea*	*Phaeodactylum tricornutum*	*Autumnella lusatica*	*Botryococcus braunii*	*Chlorococcum novae-angliae*	*Klebsormidium* sp.	*Myrmecia bisecta*	*Spongiochloris minor*	*Stichococcus* sp.	*Tetradesmus obliquus*	*Tetraselmis suecica*		*Chlorococcum novae-angliae*	*Microchloropsis salina*	*Tetradesmus obliquus*	*Spongiochloris minor*	
Kingdom	*Cr*						*Pl*										*Pl*	*Cr*	*Pl*	*Pl*	
CP	SP	SP	SP	SP	SP	SP	SP	SP	SP	SP	SP	SP	SP	SP	SP	◊	GP	GP	GP	GP	O
Alanine	1.89	1.26	0.77 ± 0.15	1.32	1.90 ± 0.04	1.28	1.95	1.06 ± 0.07	1.43 ± 0.02	1.97	2.66	2.52 ± 0.14	1.156 ± 0.002	1.71 ± 0.05	0.80	*0.38*	2.92 ± 0.37	2.56 ± 0.14	1.71 ± 0.05	2.03 ± 0.10	*0.12*
Arginine	1.34	0.97	0.57 ± 0.10	1.00	1.25 ± 0.03	0.89	1.54	1.14 ± 0.07	0.92 ± 0.01	1.37	1.32	3.18 ± 0.18	0.81 ± 0.01	0.88 ± 0.02	0.53	*0.54*	2.14 ± 0.26	2.03 ± 0.14	3.03 ± 0.08	1.27 ± 0.04	*0.04*
Aspartic acid	2.20	1.62	0.99 ± 0.17	1.58	2.65 ± 0.04	1.78	2.24	1.35 ± 0.08	1.77 ± 0.02	2.49	2.63	3.09 ± 0.21	1.22 ± 0.01	1.72 ± 0.05	0.99	*0.73*	3.59 ± 0.50	3.43 ± 0.03	1.94 ± 0.07	1.84 ± 0.18	*0.16*
Cysteine	0.04	0.02	0.029 ± 0.004	0.03	0.039 ± 0.003	0.01	0.01	0.06 ± 0.03	0.114 ± 0.001	0.01	0.04	0.12 ± 0.05	0.021 ± 0.001	0.081 ± 0.001	<LOQ	*0.25*	0.26 ± 0.05	0.06 ± 0.01	0.08 ± 0.0.1	0.06 ± 0.01	*0.78*
Glutamic acid	2.95	1.93	1.00 ± 0.25	2.04	2.63 ± 0.05	2.08	2.69	1.58 ± 0.10	1.74 ± 0.01	3.37	3.01	3.26 ± 0.19	1.44 ± 0.01	2.05 ± 0.04	1.20	*0.79*	3.61 ± 0.39	3.37 ± 0.03	2.11 ± 0.01	2.31 ± 0.12	*0.12*
Glycine	1.35	1.05	0.63 ± 0.12	1.04	1.77 ± 0.03	1.01	1.55	0.86 ± 0.05	1.03 ± 0.01	1.57	1.75	1.80 ± 0.10	0.83 ± 0.01	1.19 ± 0.05	0.62	*0.84*	2.14 ± 0.28	2.01 ± 0.12	1.24 ± 0.04	1.47 ± 0.06	*0.12*
Histidine	0.38	0.31	0.28 ± 0.06	0.08	0.40 ± 0.01	0.07	0.51	0.28 ± 0.02	0.29 ± 0.	0.49	0.58	0.42 ± 0.06	0.054 ± 0.001	0.30 ± 0.01	0.19	*0.89*	0.69 ± 0.07	0.81 ± 0.13	0.42 ± 0.04	0.36 ± 0.02	*0.02*
**Isoleucine**	1.02	0.73	0.50 ± 0.08	0.72	1.08 ± 0.02	0.78	0.94	0.64 ± 0.04	0.79 ± 0.02	0.95	1.04	1.21 ± 0.07	0.516 ± 0.003	0.78 ± 0.03	0.44	*0.95*	1.56 ± 0.19	1.62 ± 0.13	0.80 ± 0.03	0.93 ± 0.01	*0.12*
**Leucine**	2.11	1.40	0.93 ± 0.16	1.53	1.91 ± 0.03	1.41	2.19	1.38 ± 0.09	1.63 ± 0.02	2.18	2.31	2.48 ± 0.17	1.14 ± 0.01	1.62 ± 0.05	0.89	*0.31*	3.21 ± 0.41	3.06 ± 0.22	1.65 ± 0.05	2.02 ± 0.07	*0.12*
**Lysine**	1.24	1.14	0.56 ± 0.10	1.15	1.21 ± 0.02	1.02	1.55	0.89 ± 0.06	1.07 ± 0.01	1.43	1.37	1.81 ± 0.07	0.907 ± 0.001	1.03 ± 0.05	0.67	*0.79*	2.19 ± 0.26	1.76 ± 0.10	1.29 ± 0.07	1.28 ± 0.01	*0.12*
**Methionine**	0.45	0.10	0.03 ± 0.01	0.17	0.18 ± 0.08	0.32	0.33	0.07 ± 0.02	0.04 ± 0.01	0.36	0.18	0.26 ± 0.07	0.10 ± 0.02	0.02 ± 0.02	0.32	*0.79*	0.07 ± 0.01	0.19 ± 0.05	0.086 ± 0.003	0.038 ± 0.001	*0.91*
**Phenylalanine**	1.34	0.84	0.66 ± 0.06	0.89	1.36 ± 0.03	1.09	1.52	0.86 ± 0.06	1.08 ± 0.01	1.46	1.54	1.57 ± 0.11	0.67 ± 0.01	1.06 ± 0.03	0.58	*0.59*	2.13 ± 0.23	1.90 ± 0.14	1.04 ± 0.03	1.37 ± 0.05	*0.12*
Proline	1.15	0.82	0.55 ± 0.04	0.78	1.15 ± 0.01	0.67	1.34	0.79 ± 0.03	1.26 ± 0.30	1.47	1.43	1.50 ± 0.09	0.95 ± 0.23	1.27 ± 0.28	0.49	*0.05*	2.08 ± 0.47	1.93 ± 0.40	1.32 ± 0.29	1.21 ± 0.09	*0.26*
Serine	1.22	0.78	0.48 ± 0.10	0.86	1.48 ± 0.03	1.00	1.24	0.64 ± 0.04	0.92 ± 0.01	1.29	1.16	1.45 ± 0.10	0.68 ± 0.01	0.88 ± 0.01	0.56	*0.99*	1.81 ± 0.19	1.51 ± 0.07	0.917 ± 0.003	1.04 ± 0.07	*0.16*
**Threonine**	1.18	0.90	0.58 ± 0.11	0.83	1.36 ± 0.03	0.89	1.19	0.75 ± 0.05	1.000 ± 0.001	1.35	1.32	1.71 ± 0.11	0.80 ± 0.01	1.11 ± 0.02	0.57	*0.59*	1.95 ± 0.23	1.77 ± 0.10	1.18 ± 0.03	1.26 ± 0.06	*0.12*
**Tryptophan**	0.18	0.09	0.101 ± 0.004	<LOQ	0.14 ± 0.01	0.04	0.27	0.075 ± 0.001	0.089 ± 0.007	0.32	0.04	0.16 ± 0.02	0.022 ± 0.02	0.084 ± 0.004	0.03	*0.54*	0.16 ± 0.03	0.20 ± 0.05	0.115 ± 0.002	0.11 ± 0.01	*0.12*
Tyrosine	0.66	0.57	0.25 ± 0.03	0.64	1.11 ± 0.03	0.72	1.02	0.49 ± 0.01	0.15 ± 0.02	1.08	1.02	0.67 ± 0.06	0.50 ± 0.01	0.20 ± 0.01	0.38	*0.22*	0.69 ± 0.01	0.83 ± 0.02	0.41 ± 0.02	0.43 ± 0.04	*0.09*
**Valine**	1.35	0.98	0.65 ± 0.10	0.96	1.31 ± 0.03	0.96	1.39	0.84 ± 0.06	1.14 ± 0.02	1.32	1.45	1.85 ± 0.10	0.721 ± 0.005	1.17 ± 0.03	0.65	*0.54*	2.19 ± 0.28	2.02 ± 0.16	1.20 ± 0.05	1.31 ± 0.02	*0.16*
Taurine	0.13	0.18	0.05 ± 0.01	0.08	0.039 ± 0.001	0.12	0.07	0.019 ± 0.003	0.19 ± 0.01	0.09	0.08	0.202 ± 0.002	0.106 ± 0.002	0.14 ± 0.04	0.20	*0.17*	0.09 ± 0.02	0.054 ± 0.001	0.194 ± 0.005	0.079 ± 0.003	*0.16*
S-adenosyl-methionine	<LOQ	<LOQ	0.01 ± 0.01	0.06	0.018 ± 0.005	0.05	0.06	<LOQ	0.07 ± 0.01	0.01	0.08	0.07 ± 0.02	0.05 ± 0.05	0.029 ± 0.003	<LOQ	*0.17*	0.04 ± 0.01	0.03 ± 0.01	0.01 ± 0.01	<LOQ	*0.09*
Cystine	0.40	0.17	0.08 ± 0.01	0.18	0.197 ± 0.001	0.12	0.16	0.11 ± 0.01	0.11 ± 0.01	0.19	0.29	0.21 ± 0.04	0.127 ± 0.006	0.10 ± 0.01	0.12	*0.45*	0.16 ± 0.02	0.16 ± 0.06	0.13 ± 0.01	0.11 ± 0.02	*0.67*
γ-amino-butyric acid	0.03	<LOQ	0.13 ± 0.03	<LOQ	0.037 ± 0.001	0.02	0.47	0.024 ± 0.005	0.03 ± 0.01	0.03	0.03	0.08 ± 0.03	<LOQ.	0.500 ± 0.001	0.01	*0.71*	0.062 ± 0.002	0.23 ± 0.02	0.06 ± 0.01	0.07 ± 0.01	*0.67*
Ornithine	0.04	0.02	0.02 ± 0.01	0.03	0.151 ± 0.001	0.04	0.05	0.016 ± 0.002	0.033 ± 0.003	0.02	0.09	0.061 ± 0.001	0.013 ± 0.001	0.029 ± 0.002	<LOQ	*0.38*	0.07 ± 0.01	0.12 ± 0.01	0.08 ± 0.01	0.027 ± 0.001	*0.03*
NH_4_^+^	0.41	0.36	0.23 ± 0.03	0.35	0.52 ± 0.01	0.00	0.00	0.26 ± 0.03	0.49 ± 0.03	0.39	0.54	0.73 ± 0.01	0.275 ± 0.001	0.41 ± 0.02	0.20	*0.52*	0.88 ± 0.07	0.69 ± 0.06	0.51 ± 0.04	0.52 ± 0.02	*0.26*
*Sum*																					
NEA	11.46	8.05	4.70 ± 0.65	8.27	12.72 ± 0.28	8.56	12.04	6.83 ± 0.39	8.42 ± 0.35	13.25	13.71	14.41 ± 0.64	6.80 ±0.24	9.10 ± 0.42	5.03	*0.48*	17.10 ± 1.22	15.71 ± 0.68	9.74 ± 0.43	10.39 ± 0.51	*0.27*
SEA	1.72	1.27	0.86 ± 0.11	1.08	1.65 ± 0.03	0.97	2.05	1.42 ± 0.08	1.21 ± 0.01	1.85	1.90	3.60 ± 0.19	0.87 ± 0.01	1.18 ±0.02	0.71	*0.35*	2.83 ± 0.27	2.84 ±0.19	3.46 ± 0.09	1.63 ± 0.04	*0.14*
EAA	8.89	6.20	4.11 ± 0.26	6.25	8.54 ± 0.11	6.52	9.38	5.51 ± 0.15	6.84 ± 0.04	9.36	9.25	11.05 ± 0.28	4.87 ± 0.02	6.89 0.09	4.15	*0.35*	13.46 ± 0.68	12.52 ± 0.37	7.36 ± 0.12	8.32 ± 0.11	*0.27*
N-factor	4.83	5.46	4.38	4.67	4.66	5.47	5.14	4.56	4.42	4.76	4.89	5.61	4.84	4.65	4.81	*0.99*	4.77	4.77	4.24	3.93	*0.47*

Cr, chromista; CP, cultivation phase; EAA, essential amino acids (printed in bold); GP, growth phase; LOQ, limit of quantification (LOQ < 0.001 g/100 g dry weight); NEA, non-essential amino acids; Pl, plantae; SEA, semi-essential amino acids; SP, stationary phase; ◊, *p*-values between chromista and plantae of the stationary phase; O, *p*-values between the stationary and growth phase of four microalgae. Values are expressed as means ± standard deviation (*n* = 2); single determination was performed with microalgae low in biomass.

**Table 2:** Contents of nitrogenous compounds in g/100 g dry weight of 15 microalgae species in different cultivation phases.

Species	*Chrysotila carterae*	*Eustigmatos* sp.	*Microchloropsis salina*	*Nannochloropsis limnetica*	*Nitzschia palea*	*Phaeodactylum tricornutum*	*Autumnella lusatica*	*Botryococcus braunii*	*Chlorococcum novae-angliae*	*Klebsormidium* sp.	*Myrmecia bisecta*	*Spongiochloris minor*	*Stichococcus* sp.	*Tetradesmus obliquus*	*Tetraselmis suecica*		*Chlorococcum novae-angliae*	*Microchloropsis salina*	*Tetradesmus obliquus*	*Spongiochloris minor*	
Kingdom	*Cr*						*Pl*										*Pl*	*Cr*	*Pl*	*Pl*	
CP	SP	SP	SP	SP	SP	SP	SP	SP	SP	SP	SP	SP	SP	SP	SP	◊	GP	GP	GP	GP	O
Nitrogen	4.71 ± 0.06	2.41 ± 0.07	2.44 ± 0.01	3.49 ± 0.01	5.14 ± 0.03	3.05 ± 0.01	3.91 ± 0.05	3.20 ± 0.02	3.91 ± 0.05	5.56 ± 0.07	4.96 ± 0.06	4.05 ± 0.03	2.55 ± 0.01	3.77 ± 0.07	2.12 ± 0.01	*0.41*	6.72 ± 0.05	6.44 ± 0.02	6.79 ± 0.10	4.83 ± 0.02	*0.29*
NPN	0.83 ± 0.06	0.15 ± 0.08	0.14 ± 0.09	0.43 ± 0.09	0.78 ± 0.03	0.13 ± 0.03	0.83 ± 0.12	0.43 ± 0.04	0.54 ± 0.06	0.71 ± 0.19	0.84 ± 0.07	0.30 ± 0.03	0.23 ± 0.03	0.50 ± 0.13	0.53 ± 0.05	*0.58*	1.04 ± 0.13	0.54 ± 0.02	0.40 ± 0.13	1.04 ± 0.11	*0.99*
Crude protein (N-Factor 6.25)	29.4 ± 0.4	15.10 ± 0.5	15.23 ± 0.04	21.80 ± 0.05	32.1 ± 0.2	19.06 ± 0.08	30.4 ± 0.8	20.0 ± 0.1	24.4 ± 0.3	34.7 ± 0.5	31.0 ± 0.4	25.3 ± 0.2	15.94 ± 0.07	23.6 ± 0.5	13.23 ± 0.09	*0.41*	42.0 ± 0.3	40.2 ± 0.1	42.4 ± 0.6	30.2 ± 0.1	*0.29*
Crude protein (N-Factor 4.97)	23.4 ± 0.3	12.0 ± 0.4	12.11 ± 0.03	17.34 ± 0.04	25.5 ± 0.2	15.15 ± 0.06	30.4 ± 0.8	15.89 ± 0.08	19.4 ± 0.3	27.6 ± 0.4	24.6 ± 0.3	20.1 ± 0.2	12.67 ± 0.06	18.7 ± 0.4	10.52 ± 0.07	*0.41*	33.4 ± 0.3	32.00 ± 0.08	33.7 ± 0.5	24.0 ± 0.1	*0.29*
Crude protein (Specific N-Factor)	22.7 ± 0.3	13.2 ± 0.4	10.67 ± 0.03	16.29 ± 0.04	24.0 ± 0.2	16.7 ± 0.1	25.0 ± 0.6	14.6 ± 0.1	17.3 ± 0.2	26.4 ± 0.4	24.2 ± 0.3	22.7 ± 0.2	12.3 ± 0.1	17.5 ± 0.3	10.2 ± 0.1	*0.29*	32.0 ± 0.3	30.7 ± 0.1	20.5 ± 0.1	26.7 ± 0.4	*0.04*
Pure protein (Specific N-Factor)	18.7 ± 0.1	12.3 ± 0.2	10.0 ± 0.4	14.3 ± 0.4	20.31 ± 0.01	16.0 ± 0.2	20.68 ± 0.01	12.6 ± 0.2	14.9 ± 0.2	23.1 ± 0.8	20.1 ± 0.2	21.02 ± 0.04	11.2 ± 0.2	15.2 ± 0.5	7.7 ± 0.2	*0.45*	27.1 ± 0.5	28.16 ± 0.01	16.0 ± 0.5	25.1 ± 0.3	*0.04*
Total fiber (Specific N-Factor)	41.2 ± 1.1	34.2 ± 0.2	21.0 ± 1.2	14.3 ± 0.1	20.6 ± 0.7	29.4 ± 0.4	29.4 ± 0.4	34.0 ± 1.6	25.1 ± 2.1	28.9 ± 0.7	12.7 ± 0.5	40.5 ± 2.2	28.0 ± 0.3	34.6 ± 2.4	27.5 ± 1.3	*0.68*	32.6 ± 1.0	23.8 ± 0.8	36.6 ± 0.7	40.4 ± 0.5	*0.27*

Cr, chromista; CP, cultivation phase; GP, growth phase; NPN, non-protein nitrogen; Pl, plantae; SP, stationary phase; ◊, *p*-values between chromista and plantae of the stationary phase; O, *p*-values between the stationary and growth phase of four microalgae. Values are expressed as means ± standard deviation (*n* = 2).

### 2.3. Total Fat and Fatty Acids

The total fat content of microalgal biomass in the growth phase varied from 6.8 (*C. novae-angliae*) to 15.3 g/100 g (*M. salina*), while in the stationary phase, it ranged from 5.9 (*C. novae-angliae*) to 53.0 g/100 g (*M. salina*). The total fat content as well as the fatty acid profile of microalgal biomass in the growth phase versus the stationary phase did not differ significantly (Table 3). In chromista, the total fat, ranging from 8.6 (*N. palea*) to 53.0 g/100 g (*M. salina*), was higher than in plantae, ranging from 4.9 (*Klebsormidium* sp.) to 51.1 g/100 g (*B. braunii*; *p* < 0.05). Comparing both kingdoms, the contents of total fat, saturated fatty acids (SFAs), C14:0, C16:1_n7_, C20:4_n6_ and C20:5_n3_ were higher in chromista than in plantae (*p* < 0.05, Table 3).

Furthermore, the SFA content in chromista, between 2.3 (*N. palea*) and 26.8 g/100 g (*M. salina*), was higher than the contents in plantae, ranging from 1.2 (*M. bisecta*) to 14.3 g/100 g (*B. braunii*; *p* < 0.05). The SFA content of biomass in the growth phase ranged from 1.3 (*S. minor*) to 4.9 g/100 g (*M. salina*), while in the stationary phase, it ranged from 1.6 (*T. obliquus*) to 26.8 g/100 g (*M. salina*). 

The monounsaturated fatty acid (MUFAs) content in chromista varied from 3.3 (*N. palea*) to 22.3 g/100 g (*Eustigmatos* sp.) and in plantae from 0.3 (*Klebsormidium* sp.) to 20.1 g/100 g (*Botryococcus* sp.). The MUFA content in biomass in the growth phase ranged from 0.8 (*S. minor*) to 4.1 g/100 g (*M. salina*), while in the stationary phase, it varied from 1.2 (*C. novae-angliae*) to 20.5 g/100 g (*M. salina*). 

The contents of PUFAs in chromista ranged from 1.6 (*N. palea*) to 4.2 g/100 g (*P. tricornutum*) and in plantae, it ranged from 0.9 (*Klebsormidium* sp.) to 9.5 g/100 g (*B. braunii*). The PUFA content in biomass in the growth phase varied from 1.9 (*T. obliquus*) to 3.6 g/100 g (*M. salina*), while in the stationary phase, it ranged from 1.6 (*C. novae-angliae*) to 3.2 g/100 g (*M. salina*). 

The C18:3_n3_ content of chromista with a range from below the limit of quantification (LOQ, <0.1 g/100 g d.w. *N. limnetica)* to 1.23 g/100 g (*C. carterae)* was lower than the contents in plantae, which were between 0.07 (*Klebsormidium* sp.) and 6.7 g/100 g (*B. braunii*; *p* < 0.05). C18:3_n3_ was the dominant n3-PUFA in plantae. The contents of C18:3_n3_ were not different between the growth phase, ranging from <LOQ (*M. salina*) to 2.59 g/100 g (*S. minor*), and stationary phase, ranging from 0.01 (*M. salina*) to 1.34 g/100 g (*T. obliquus*). 

C20:5_n3_ was the dominant n3-PUFA in chromista, with contents between 0.25 (*C. carterae*) and 2.78 g/100 g (*P. tricornutum*), which is higher than that of plantae, ranging from <LOQ (*A. lusatica* and *C. novae-angliae*) to 1.08 g/100 g (*B. braunii*; *p* < 0.05). In the growth phase, C20:5_n3_ contents ranged from <LOQ (*S. minor*) to 2.97 g/100 g (*M. salina*), while the range in the stationary phase was from <LOQ (*C. novae-angliae*) to 1.7 g/100 g (*M. salina*). 

The n6/n3 PUFA ratios ranged from 0.4 (*P. tricornutum)* to 1.6 (*N. palea*) in the chromista kingdom and from 0.2 (*B. braunii*) to 9.15 (*Klebsormidium* sp.) in plantae (Table 3). The range of the n6/n3 PUFA ratio in the growth phase was from 0.22 (*M. salina*) to 2.41 (*C. novae-angliae*), whereas it ranged from 0.64 (*T. obliquus*) to 2.18 (*C. novae-angliae*) in the stationary phase.

Additional parameters shown in Table 3 were comparable between kingdoms and cultivation phases, respectively.

**Table 3:** Total fat content and fatty acid composition in g/100 g dry weight of 15 microalgae species in different cultivation phases.

Species	*Chrysotila carterae*	*Eustigmatos* sp.	*Microchloropsis salina*	*Nannochloropsis limnetica*	*Nitzschia palea*	*Phaeodactylum tricornutum*	*Autumnella lusatica*	*Botryococcus braunii*	*Chlorococcum novae-angliae*	*Klebsormidium* sp.	*Myrmecia bisecta*	*Spongiochloris minor*	*Stichococcus* sp.	*Tetradesmus obliquus*	*Tetraselmis suecica*		*Chlorococcum novae-angliae*	*Microchloropsis salina*	*Spongiochloris minor*	*Tetradesmus obliquus*	
Kingdom	*Cr*						*Pl*										*Pl*	*Cr*	*Pl*	*Pl*	
CP	**SP**	**SP**	**SP**	**SP**	**SP**	**SP**	**SP**	**SP**	**SP**	**SP**	**SP**	**SP**	**SP**	**SP**	**SP**	◊	**GP**	**GP**	**GP**	**GP**	O
Total fat	12.9 ± 0.1	38.3 ± 0.2	53.0 ± 0.3	40.1 ± 0.3	8.64 ± 0.09	27.43 ± 0.04	20.6 ± 0.6	51.09 ± 0.54	5.85 ± 0.18	4.91 ± 0.11	6.22 ± 0.27	8.93 ± 0.06	33.87 ± 0.08	8.07 ± 0.19	9.21 ± 0.14	*0.03*	6.78 ± 0.23	15.3 ± 0.3	7.71 ± 0.10	7.66 ± 0.22	*0.17*
***SFA***																					
C14:0	0.031	1.21	3.23	1.90	0.45	2.16	0.037	0.44	0.17	0.054	0.010	0.069	0.33	0.022	0.028	*0.01*	0.15	1.10	0.015	0.019	*0.37*
C16:0	3.34	10.9	22.8	13.4	1.65	7.72	3.91	11.69	1.22	1.88	1.11	1.59	8.01	1.50	2.26	*0.06*	1.57	3.48	1.09	1.41	*0.27*
C18:0	0.16	0.52	0.56	0.65	0.065	0.19	0.35	0.64	0.26	0.14	0.044	0.26	0.81	0.062	0.047	*0.64*	0.19	0.15	0.14	0.076	*0.14*
C20:0	<LOQ	0.022	<LOQ	0.071	0.005	0.019	0.065	0.93	0.029	0.021	0.006	0.052	0.14	<LOQ	<LOQ	*0.29*	0.017	0.013	0.014	0.004	*0.47*
C22:0	<LOQ	<LOQ	<LOQ	<LOQ	0.009	0.021	0.10	0.52	0.012	0.15	0.006	0.016	0.075	0.020	<LOQ	*0.03*	0.006	0.001	0.006	0.018	*0.14*
C24:0	0.011	<LOQ	<LOQ	<LOQ	0.070	0.11	0.33	<LOQ	0.016	0.037	0.061	0.049	0.20	0.012	0.007	*0.26*	0.007	0.001	0.023	0.013	*0.47*
***MUFA***																					
C16:1_n7_	0.36	18.8	13.3	10.4	2.85	9.15	0.063	2.71	0.078	0.068	0.047	0.29	0.034	0.058	0.037	*0.002*	0.067	3.31	0.033	0.060	*0.38*
C17:1_n7_	<LOQ	<LOQ	0.15	<LOQ	<LOQ	<LOQ	<LOQ	<LOQ	0.003	0.032	0.014	0.016	<LOQ	0.033	<LOQ	*X*	0.008	0.078	0.014	0.030	*0.47*
C18:1_n9_	2.82	3.34	6.98	9.08	0.053	1.84	5.29	15.8	0.14	0.16	0.99	3.38	13.6	1.55	2.46	*0.91*	0.19	0.63	0.65	1.39	*0.14*
C18:1_n7_	0.39	0.24	<LOQ	0.17	0.41	0.13	0.17	0.98	0.97	<LOQ	0.40	0.063	0.083	0.066	0.38	*0.95*	0.85	0.058	0.070	0.063	*0.70*
C20:1_n9_	<LOQ	<LOQ	0.002	0.030	<LOQ	<LOQ	0.065	0.58	0.014	<LOQ	0.017	0.050	0.26	0.015	0.12	*0.02*	0.015	0.005	0.029	0.014	*0.72*
***n6-PUFA***																					
C18:2_n6_	0.88	0.83	0.33	0.95	0.026	0.29	2.93	1.69	0.66	0.72	0.83	1.33	4.44	0.78	0.94	*0.10*	1.22	0.12	0.86	0.67	*0.72*
C18:3_n6_	0.049	0.042	0.11	0.064	0.040	0.20	0.170	<LOQ	0.44	0.028	0.018	0.034	0.080	0.078	0.069	*0.64*	0.48	0.031	0.023	0.067	*0.47*
C20:2_n6_	0.039	<LOQ	<LOQ	0.028	<LOQ	0.026	<LOQ	<LOQ	0.005	0.010	0.021	0.001	0.15	<LOQ	0.012	*0.86*	0.004	0.004	0.007	0.001	*0.47*
C20:3_n6_	0.014	0.089	<LOQ	0.10	0.009	0.032	<LOQ	<LOQ	<LOQ	0.026	0.020	0.002	0.25	<LOQ	0.017	*0.28*	<LOQ	0.032	<LOQ	<LOQ	*X*
C20:4_n6_	0.043	0.15	1.01	0.77	0.89	0.70	<LOQ	<LOQ	<LOQ	0.030	0.68	0.019	0.50	0.001	0.21	*0.01*	<LOQ	0.47	<LOQ	0.001	*0.37*
***n3-PUFA***																					
C18:3_n3_	1.23	0.21	0.009	<LOQ	0.068	0.023	3.25	6.70	0.51	0.076	0.44	0.74	2.87	1.34	0.46	*0.01*	0.71	<LOQ	2.59	1.10	*0.72*
C20:5_n3_	0.25	1.24	1.70	1.68	0.51	2.78	<LOQ	1.08	<LOQ	0.015	0.091	0.04	0.10	0.004	0.50	*0.005*	0.001	2.97	<LOQ	0.008	*0.41*
C22:6_n3_	0.86	<LOQ	<LOQ	<LOQ	0.044	0.15	0.014	<LOQ	<LOQ	<LOQ	<LOQ	<LOQ	<LOQ	<LOQ	<LOQ	*X*	<LOQ	<LOQ	<LOQ	<LOQ	*X*
***Sum***																					
SFA	3.54	12.6	26.8	16.1	2.26	10.2	4.79	14.3	1.70	2.28	1.23	2.04	9.56	1.62	2.34	*0.045*	1.93	4.86	1.29	1.54	*0.27*
MUFA	3.56	22.3	20.5	19.8	3.31	11.1	5.59	20.1	1.21	0.25	1.46	3.80	14.0	1.72	3.00	*0.06*	1.13	4.10	0.79	1.55	*0.07*
PUFA	3.51	2.57	3.15	3.59	1.59	4.19	6.37	9.47	1.62	0.93	2.10	2.17	8.40	2.20	2.21	*0.64*	2.41	3.64	3.48	1.85	*0.14*
n6-PUFA	1.16	1.12	1.45	1.92	0.97	1.24	3.10	1.69	1.11	0.84	1.56	1.39	5.43	0.85	1.25	*0.48*	1.70	0.66	0.89	0.74	*0.47*
n3-PUFA	2.35	1.46	1.71	1.68	0.62	2.95	3.27	7.78	0.51	0.09	0.54	0.78	2.97	1.34	0.96	*0.64*	0.71	2.97	2.59	1.11	*0.27*
n6/n3	0.50	0.77	0.85	1.14	1.55	0.42	0.95	0.22	2.18	9.15	2.92	1.77	1.83	0.64	1.30	*0.48*	2.41	0.22	0.34	0.66	*0.47*
Others	2.28	0.77	2.51	0.67	1.48	1.87	3.81	7.22	1.32	1.44	1.43	0.91	1.91	2.53	1.66	*0.10*	1.31	2.72	2.15	2.72	*0.14*

Cr, chromista; CP, cultivation phase; GP, growth phase; LOQ, limit of quantification; MUFA, monounsaturated fatty acids; Pl, plantae; PUFA, poly-unsaturated fatty acids; SFA, saturated fatty acids; SP, stationary phase; LOQ < 0.1 g/100 g dry weight; ◊, *p*-values between chromista and plantae of the sta-tionary phase; O, *p*-values between the stationary and growth phase of four microalgae; values of total fat are expressed as means ± standard deviation (*n* = 2).

### 2.4. Total Carotenoids and Total Chlorophyll

**Figure 1:**
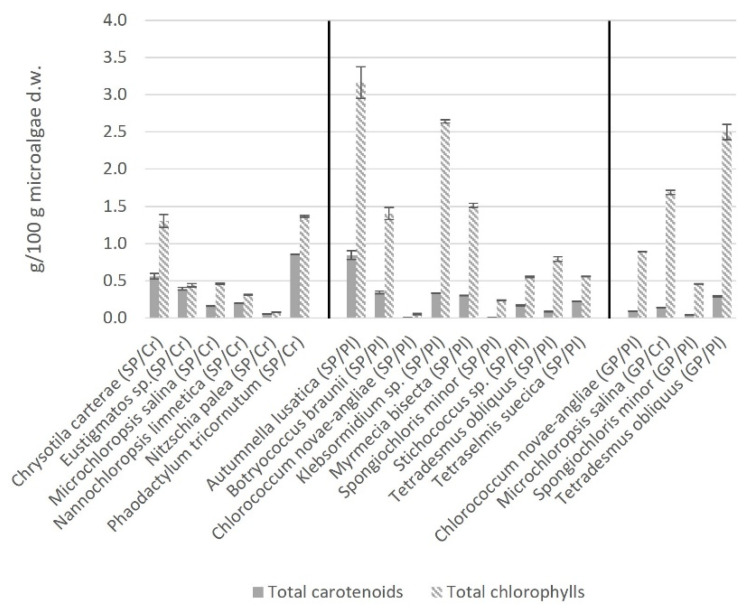
Concentration of total carotenoids and total chlorophyll of fifteen microalgae in g/100 g dry weight. There were no significant differences between kingdoms or between cultivation phases (*p* > 0.05; *n* = 3; Cr, chromista; GP, growth phase; Pl, plantae; SP, stationary phase).

Regarding the concentration of total carotenoids and total chlorophyll, there were no significant differences detected between both kingdoms or both cultivation phases (Figure 1). 

The total carotenoid concentrations ranged from 0.05 (*N. palea*) to 0.86 g/100 g (*P. tricornutum*) in chromista and from 0.01 (*C. novae-angliae* and *S. minor*) to 0.84 g/100 g (*A. lusatica*) in plantae. The ranges regarding both cultivation phases were from 0.04 (*S. minor*) to 0.29 g/100 g (*T. obliquus*) in the growth phase and from 0.01 (*C. novae-angliae* and *S. minor*) to 0.16 g/100 g (*M. salina*) in the stationary phase. 

### 2.5. Main Elements 

The main elements C, H, N and S were not significantly different between the microalgae biomass of both kingdoms or cultivation phases (Table 4; for N, see also Table 2). 

Carbon was the most abundant element in the studied microalgae (Table 4). In chromista, the C concentration ranged from 25 (*N. palea*) to 59 g/100 g d.w. (*M. salina*) and in plantae from 35 (*M. bisecta*) to 68 g/100 g d.w. (*B. braunii*). In the growth phase, the range was from 42 *(S. minor*) to 49 g/100 g d.w. (*M. salina*) and in the stationary phase from 44 (*S. minor*) to 59 g/100 g d.w. (*M. salina*). 

### 2.6. Minerals, Trace Elements and Heavy Metals 

The contents of Ca and As in chromista were higher than those in plantae, whereas the content of Pb was higher in plantae (*p* < 0.05; Table 4). Furthermore, the contents of Mg, Mn, Fe and Zn in biomass in the growth phase were higher than that in the stationary phase. However, the contents of Ni, Mo and I_2_ were higher in the stationary phase compared to the growth phase (*p* < 0.05). 

The Mg content in chromista ranged from 147 (*N. limnetica*) to 496 mg/100 g d.w. (*C. carterae*) and in plantae from 74 (*B. braunii*) to 1065 mg/100 g d.w. (*M. bisecta;* Table 4). The Mg content in the growth phase, ranging from 169 (*S. minor*) to 333 mg/100 g d.w. (*M. salina*), was higher than that in the stationary phase, ranging from 189 (*S. minor*) to 226 mg/100 g d.w. (*T. obliquus*, *p* < 0.05). Higher Ca contents were detected in chromista, with a range from 22 (*N. palea*) to 286 mg/100 g d.w. (*p. tricornutum*), compared to plantae, with a range from 15 (*B. braunii*) to 1707 mg/100 g d.w. (*T. suecica; p* < 0.05). During the growth phase, Ca contents ranged from 115 (*S. minor*) to 197 mg/100 g d.w. (*M. salina*) and during stationary phase from 80 (*M. salina*) to 233 mg/100 g d.w. (*T. obliquus*). 

Among the analyzed trace elements, iron was the dominant one detected in all microalgae. The Fe content in the chromista microalgae ranged from 71 (*M. salina*) to 441 mg/100 g d.w. (*N. palea*) and in plantae from 90 (*B. braunii*) to 1359 mg/100 g d.w. (*M. bisecta*; Table 4). The Fe content of growing biomass, ranging from 99 (*T. obliquus*) to 205 mg/100 g d.w. (*C. novae-angliae*), was higher than the Fe content in stationary biomass, ranging from 71 (*M. salina*) to 135 mg/100 g d.w. (*C. novae-angliae*; *p* < 0.05). The Zn content in the chromista microalgae ranged from 1.5 (*N. limnetica*) to 5.5 mg/100 g d.w. (*C. carterae*) and in plantae from 0.5 (*T. obliquus*) to 2.6 mg/100 g d.w. (*M. bisecta;* Table 4). The Zn content in the growth phase, ranging from 0.7 (*C. novae-angliae* and *S. minor*) to 3.2 mg/100 g d.w. (*M. salina*), was higher compared to that in the stationary phase, ranging from 0.5 (*T. obliquus*) to 1.6 mg/100 g d.w. (*M. salina*; *p* < 0.05). The I_2_ content in the chromista microalgae ranged from 12 (*N. limnetica*) to 840 µg/100 g d.w. (*C. carterae*) and in plantae from 8 (*B. braunii*) to 70 µg/100 g d.w. (*Stichococcus* sp.; Table 4). Differences were determined concerning the content of I_2_, with lower content in the growth phase, ranging from 33 (*C. novae-angliae* and *T. obliquus*) to 72 µg/100 g d.w. (*M. salina*), compared to the stationary phase, ranging from 39 (*T. obliquus*) to 72 µg/100 g d.w. (*M. salina*, *p* < 0.05). 

The content of As in chromista was higher than that in plantae (*p* < 0.05). The former ranged from 8.6 (*Eustigmatos* sp.) to 129.0 µg/100 g d.w. (*M. salina*), whereas the latter ranged from 2.7 (*T. obliquus*) to 37.4 µg/100 g d.w. (*M. bisecta*). In the growth phase, the As content ranged from 2.1 (*T. obliquus*) to 234.0 µg/100 g d.w. (*M. salina*) and in the stationary phase from 2.7 (*T. obliquus*) to 129.0 µg/100 g d.w. (*M. salina*). The content of Pb was lower in chromista in comparison to plantae (*p* < 0.05), ranging from 0.1 (*M.*) to 15.2 mg/100 g d.w. (*N. limnetica*) in chromista and 0.8 (*B. braunii*) to 11.3 mg/100 g d.w. (*T. suecica*) in plantae. While the range in the growth phase was from 0.2 (*M. salina*) to 2.3 mg/100 g d.w. (*C. novae-angliae*), in the stationary phase, it was from 0.1 (*M. salina*) to 3.4 mg/100 g d.w. (*C. novae-angliae*).

Further parameters of Table 4 were comparable between kingdoms and cultivation phases, respectively.

**Table 4:** Content of minerals and trace elements with their recommended daily intake and heavy metals with tolerable daily amounts in the dry weight of 15 microalgae species in different cultivation phases.

Species	*Chrysotila carterae*	*Eustigmatos* sp.	*Microchloropsis salina*	*Nannochloropsis limnetica*	*Nitzschia palea*	*Phaeodactylum tricornutum*	*Autumnella lusatica*	*Botryococcus braunii*	*Chlorococcum novae-angliae*	*Klebsormidium* sp.	*Myrmecia bisecta*	*Spongiochloris minor*	*Stichococcus* sp.	*Tetradesmus obliquus*	*Tetraselmis suecica*		*Chlorococcum novae-angliae*	*Microchloropsis salina*	*Spongiochloris minor*	*Tetradesmus obliquus*		RDI and TDI
Kingdom	*Cr*						*Pl*										*Pl*	*Cr*	*Pl*	*Pl*		
CP	**SP**	**SP**	**SP**	**SP**	**SP**	**SP**	**SP**	**SP**	**SP**	**SP**	**SP**	**SP**	**SP**	**SP**	**SP**	**◊**	**GP**	**GP**	**GP**	**GP**	**O**	
*Main elements*																						
**C** *(g/100 g)*	43.47 ± 0.30	54.45 ± 0.20	58.813 ± 0.005	53.97 ± 0.39	25.13 ± 0.10	48.38 ± 0.14	49.51 ± 0.51	67.99 ± 0.93	43.77 ± 0.74	42.91 ± 0.33	34.69 ± 0.41	43.59 ± 0.91	50.09 ± 0.47	44.81 ± 0.79	40.02 ± 0.14	*0.75*	43.67 ± 0.70	48.73 ± 0.26	42.49 ± 0.80	46.42 ± 1.02	0.94	X
**H_2_** *(g/100 g)*	6.23 ± 0.05	8.18 ± 0.03	9.01 ± 0.01	8.08 ± 0.02	4.06 ± 0.02	7.17 ± 0.02	7.05 ± 0.06	9.63 ± 0.18	6.60 ± 0.08	6.18 ± 0.04	5.18 ± 0.05	6.62 ± 0.17	7.45 ± 0.08	6.73 ± 0.10	6.21 ± 0.03	*0.52*	6.42 ± 0.11	7.23 ± 0.05	6.55 ± 0.14	6.74 ± 0.16	0.88	X
**S** *(g/100 g)*	0.96 ± 0.02	0.22 ± 0.04	0.32 ± 0.03	0.37 ± 0.12	0.52 ± 0.06	0.73 ± 0.04	0.55 ± 0.04	0.21 ± 0.04	0.43 ± 0.03	0.39 ±0.03	0.39 ± 0.04	0.38 ± 0.04	0.35 ±0.04	0.42 ±0.06	1.13 ±0.02	*0.65*	0.60 ±0.01	0.58 ±0.04	0.34 ±0.01	0.48 ±0.03	0.93	X
*Minerals*																						
**Mg** *(mg/100 g)*	496 ± 5	276 ± 2	219 ± 1	146.7 ± 0.5	331 ± 6	333 ± 3	334 ± 4	74.0 ± 1.6	218 ± 2	183 ± 1	1065 ± 28	188.5 ± 0.3	331 ± 3	226 ± 1	559 ± 3	*0.15*	319 ± 4	333.1 ± 0.8	169 ± 2	295 ± 18	*0.005*	300–350 mg
Ca *(mg/100 g)*	258 ± 5	240.1 ± 0.5	80.4 ± 1.4	109 ± 4	21.8 ± 2.5	268 ± 1	33.9 ±1.6	15.4 ± 0.9	188 ± 1	81.5 ± 0.2	166 ± 8	149 ± 2	70 ± 1	233 ± 3	1707 ± 19	*0.04*	182.4 ± 0.6	197 ± 3	115.0 ± 0.9	151 ± 8	*0.86*	1000 mg
*Trace elements*																						
**Mn** *(mg/100 g)*	5.82 ± 0.11	1.615 ± 0.008	1.582 ± 0.004	17.49 ± 0.03	6.23 ± 0.08	5.04 ± 0.02	3.26 ±0.01	1.96 ± 0.01	1.00 ± 0.03	1.886 ± 0.007	23.0 ± 1.5	0.76 ± 0.01	3.79 ± 0.03	0.7 ± 0.01	2.49 ± 0.01	*0.82*	1.53 ± 0.03	3.76 ± 0.02	0.90 ± 0.03	1.12 ± 0.06	*0.01*	2–5 mg
**Fe** *(mg/100 g)*	411 ± 5	247.1 ± 0.4	70.6 ± 0.5	328 ± 4	441 ± 18	217 ± 1	205.4 ± 0.9	90.3 ± 1.3	135 ± 2	203.9 ± 0.8	1359 ± 57	122 ± 2.3	214 ± 1	92 ± 3	117.8 ± 0.3	*0.33*	205 ± 6	110.7 ± 0.4	124 ± 2	98.6 ± 4.2	*0.01*	10–15 mg
**Cu** *(mg/100 g)*	1.05 ± 0.03	0.37 ± 0.01	0.290 ± 0.002	1.13 ± 0.04	3.90 ± 0.05	4.42 ± 0.01	0.447 ±0.002	0.31 ± 0.01	0.33 ± 0.01	0.633 ± 0.005	7.74 ± 0.12	0.48 ± 0.01	1.02 ± 0.01	0.380 ± 0.001	0.57 ± 0.01	*0.70*	0.150 ± 0.002	0.519 ± 0.001	0.383 ± 0.006	0.56 ± 0.02	*0.21*	1.0–1.5 mg
**Zn** *(mg/100 g)*	5.48 ± 0.09	1.68 ± 0.01	1.61 ± 0.03	1.51 ± 0.01	3.87 ± 0.06	1.60 ± 0.02	1.85 ±0.02	1.37 ± 0.02	0.77 ± 0.02	0.54 ± 0.02	2.61 ± 0.03	0.62 ± 0.06	1.22 ± 0.02	0.501 ± 0.001	1.69 ± 0.04	*0.78*	0.71 ± 0.03	3.18 ± 0.02	0.70 ± 0.01	1.26 ± 0.01	*0.02*	7–16 mg
**Se** *(μg/100 g)*	19.6 ± 2.9	10.2 ± 0.8	7.80 ± 1.03	132 ± 10	4.43 ± 0.91	3.45 ± 0.76	0.26 ±0.08	17.6 ± 1.3	7.89 ± 2.08	36.5 ± 1.4	9.19 ± 0.81	1.87 ± 0.23	20.6 ± 0.4	3.40 ± 1.10	46.1 ± 1.9	*0.08*	5.30 ± 0.12	9.86 ± 0.71	0.66 ± 0.03	2.33 ± 0.49	*0.59*	60–70 µg
**Ni** *(mg/100 g)*	12.5 ± 0.3	2.66 ± 0.01	1.11 ± 0.01	7.58 ± 0.01	7.63 ± 0.11	6.47 ± 0.05	3.65 ±0.03	2.25 ± 0.03	1.31 ± 0.04	5.45 ± 0.03	141 ± 5	0.64 ± 0.03	3.49 ± 0.01	0.494 ± 0.001	3.13 ± 0.02	*0.20*	0.41 ± 0.01	0.48 ± 0.04	0.140 ± 0.002	0.311 ± 0.006	*0.01*	0.025–0.035 mg
**Mo** *(mg/100 g)*	3.17 ± 0.42	1.363 ± 0.008	0.36 ± 0.01	3.98 ± 0.06	4.83 ± 0.25	2.02 ± 0.02	2.16 ±0.02	0.94 ± 0.06	0.74 ± 0.01	1.946 ± 0.009	5.20 ± 0.27	0.39 ± 0.02	3.08 ± 0.07	0.379 ± 0.001	1.35 ± 0.01	*0.94*	0.31 ± 0.01	0.33 ± 0.01	0.184 ± 0.001	0.275 ± 0.009	*0.01*	0.05–0.10 mg
**I_2_** *(μg/100 g)*	840 ±23	18.9 ± 5.0	72.4 ± 5.1	11.6 ± 1.5	21.6 ± 3.8	65.4 ± 8.2	39.3 ± 2.9	7.70 ± 0.4	41.2 ± 29.4	26.9 ± 5.2	20.4 ± 1.0	47.6 ± 23.8	70.0 ± 7.1	38.6 ± 16.3	62.1 ± 1.5	*0.99*	33.0 ± 27.3	71.6 ± 1.6	38.9 ± 19.0	33.3 ± 20.6	*0.02*	180–200 µg
Heavy metals ^****^																						
**As** *(μg/100 g)*	25.5 ± 1.9	8.57 ± 0.36	129 ± 2	11.5 ± 0.3	17.9 ± 0.8	59.1 ± 0.8	10.1 ± 0.2	4.74 ± 0.35	6.47 ±0.13	9.54 ± 0.17	37.4 ± 0.8	2.93 ± 0.13	9.06 ± 0.44	2.65 ± 0.03	14.7 ± 0.3	*0.01*	14.2 ± 1.1	234 ± 3	2.52 ± 0.02	2.12 ± 0.07	*0.07*	X
**Cd** *(mg/100 g)*	0.175 ± 0.003	0.191 ± 0.001	0.237 ± 0.003	2.23 ± 0.02	0.078 ± 0.001	0.016 ± 0.001	0.337 ±0.003	1.48 ± 0.01	0.38 ± 0.03	1.551 ± 0.008	0.563 ± 0.001	0.158 ± 0.06	0.117 ± 0.002	0.131 ± 0.003	1.25 ± 0.01	*0.65*	0.375 ± 0.004	0.197 ± 0.001	0.165 ± 0.009	0.113 ± 0.001	*0.05*	<0.1 mg/ 100 g
**Hg** *(μg/100 g)*	0.80 ± 0.12	0.40 ± 0.08	0.59 ± 0.07	0.62 ± 0.01	2.04 ± 0.22	0.504 ± 0.007	0.319 ±0.003	0.45 ± 0.10	1.53 ± 0.01	0.62 ± 0.04	1.58 ± 0.26	1.02 ± 0.05	0.34 ± 0.06	0.97 ± 0.13	0.38 ± 0.13	*0.53*	1.41 ± 0.23	1.16 ± 0.08	0.63 ± 0.02	0.56 ± 0.04	*0.95*	<10 μg/ 100 g
**Pb** *(mg/100 g)*	0.54 ± 0.01	0.700 ± 0.001	0.110 ± 0.005	15.23 ± 0.07	0.338 ± 0.007	0.122 ± 0.003	2.51 ±0.02	0.790 ± 0.002	3.41 ± 0.23	0.936 ± 0.007	2.20 ± 0.08	1.51 ± 0.17	1.56 ± 0.01	1.18 ± 0.18	11.26 ± 0.06	*0.006*	2.33 ± 0.18	0.167 ± 0.002	0.69 ± 0.10	0.169 ± 0.021	*0.05*	<0.3 mg/ 100 g

C, chromista; CP, cultivation phase; GP, growth phase; P, plantae; RDI, recommended daily intake; SP, stationary phase; TDI, tolerable daily intake. RDI only refers to minerals and trace elements [13,14]; TDI only refers to heavy metals [15]. ◊, *p*-values between chromista and plantae of the stationary phase; O, *p*-values between the stationary and growth phase of four microalgae. Values are expressed as means ± standard deviation (*n* = 3). **** Due to contamination of the central compressed air supply, heavy metal levels were unusually high in microalgae cultivated for this study.

## 3. Discussion

Microalgae production for human nutrition is a growing sector in the food industry. This study examined 15 hardly studied microalgae species from different kingdoms and their nutritional value. In addition, changes in the nutritional profile of four different microalgae during two different cultivation phases were investigated to evaluate their potential for human nutrition. 

### 3.1. Variability between Kingdoms and Genera

Chromista differ from plantae by the more complex membrane topology of their chloroplasts and their rigid tubular multipartite ciliary hairs [16]. The development of chloroplasts was most likely caused by an endosymbiotic event between a red alga and a heterotrophic eukaryotic host in the course of evolution [17]. Microalgae belonging to chromista are able to synthesize and accumulate larger amounts of n3-PUFA such as eicosapentaenoic acid (EPA), docosapentaenoic acid (DPA) and docosahexaenoic acid (DHA) [18]. The differences in morphology regarding the existence and location of cortical alveoli, position of chloroplasts, Golgi apparatus and phycobilisomes between chromista and plantae cause a variety in metabolites and, therefore, nutrients in different microalgae species [16]. To date, there are no data available in the literature regarding the contents of macro- and micronutrients in *Myrmecia* sp. and *Autumnella* sp.

### 3.2. Protein and Amino Acids

In the amino acid profile, only slight differences were determined among all analyzed microalgae. Microalgae synthesize all amino acids in substantial amounts but are poor in sulfur-containing amino acids, which is consistent with data from the literature [1,19]. Furthermore, the ratios between amino acids in microalgae such as *Botryococcus* sp., *Nannochloropsis* sp., *Phaeodactylum* sp. and *Tetraselmis* sp. match with those in the literature, yet the overall content does not [20]. A culture medium with a carbon to nitrogen ratio tending towards nitrogen promotes the production of amino acids [21]. We assume that a nitrogen-poorer cultivation is causing the lower amino acid concentrations in the analyzed microalgae compared to the literature. This hypothesis is supported by generally higher amino acid concentrations such as histidine and arginine in the growth phase compared to the stationary phase, where the number of microalgae is increased, which leads to competition for nutrients as well as carbon dioxide. Microalgae in the growth phase were shown to accumulate more amino acids with two or more nitrogen atoms, such as histidine and arginine but also ornithine. This might be due to a greater availability of nitrogen in the culture medium, since microalgae cultivated in a nitrogen-rich medium synthesize amino acids rich in nitrogen [21]. Furthermore, the additional increase in the non-protein amino acid ornithine is plausible, due to its role as a precursor for arginine synthesis [21]. In recent years, alternatives to animal-based protein sources have been investigated due to the higher demands for food of a continuously increasing human population [22]. Microalgae might be a potential food alternative rather than conventional plant protein sources such as lentils, beans and peas because of their protein quality and higher protein digestibility-corrected amino acid score (PDCAAS) [23]. The protein content in microalgae species such as *Chlorococcum* sp., *Microchloropsis* sp. and *Tetradesmus* sp. in the growth phase was higher than that in eggs (13%), legumes (21–26%) and beef (19%) [24]. Comparable studies described lower protein contents for *Klebsormidium* sp. [25], whereas the crude protein contents of the species *Botryococcus*, *Chlorococcum*, *Nitzschia*, *Tetradesmus* and *Stichococcus* were comparable with the literature [26,27,28,29,30,31]. The protein content of the microalgae species *Nannochloropsis*, *Phaeodactylum*, *Chrysotila*, *Spongiochloris* and *Tetraselmis* analyzed in the present study was lower than data in the literature [26,29,32,33,34,35]. Previous research into the protein content of the species *Eustigmatos*, *Autumnella* and *Myrmecia* was not found. Depending on the N-factor used for the calculation of the protein content, the protein content varies drastically and is often overestimated due to an incorrect (i.e., unspecific) N-factor [1]. It is often not considered that the N-factor between different species and cultivation phases can vary strongly [36,37]. Although the N-factor between cultivations was not different, the use of a specific calculated N-factor for each microalga caused differences in the pure and crude protein content between cultivation phases. This underlines the necessity of the usage of specific N-factors, which were obtained through amino acid analysis in this study. Furthermore, the protein content can depend on the species of microalgae, light quality and quantity, temperature, nitrogen source, nitrogen availability, CO_2_, pH and medium [38,39]. 

### 3.3. Dietary Fibers

Dietary fibers have a positive impact on human health, reducing the blood cholesterol and having an effect on digestive regulation, fecal bulk, intestinal transit time and gastric emptying [40]. Microalgae contain high amounts of fibers of great variety [41]. With an average total fiber amount of over 29%, the microalgae analyzed here have higher fiber contents than common sources such as white beans (18%), barley (17%) and soy (15%) [42]. The literature is lacking in dietary fiber references for microalgae, likely because of the high costs and time-consuming analyses. Matching contents were found for *Nannochloropsis* and *Tetradesmus*, while *Phaeodactylum* had higher total dietary fiber than that described in the literature [31,43,44]. The total fiber content in the growth phase was higher than in the stationary phase for all analyzed microalgae species except *Spongiochloris*. This is trend is supported by analysis of other microalgae described in the literature [45].

### 3.4. Lipids

The total fat content of microalgae from this study did not always match the data from the literature. Microalgae known for generally high contents of total fat are *Microchloropsis salina* (26–46%) *Nannochloropsis* sp. (22–28%)*, Phaeodactylum tricornutum* (16%), *Stichococcus bacillaris* (11–24%) and *Eustigmatos magnus* (28%) [20,46,47,48,49]. Matching fat contents were detected in the microalgae *Botryococcus*, *Chrysotila*, *Nitzschia* and *Tetraselmis* [26,28,34]. A surprising discovery is the low lipid amounts between 5 and 9% analyzed in *Klebsormidium*, *Chlorococcum* and *Spongiochloris,* while the literature indicates lipid amounts of 32, 33 and 27–46%, respectively [25,27,32,50]. During the growth of biomass, the microalgae start to synthesize lipids until a plateau is reached in their stationary phase [51]. This was not confirmed by the present data. Microalgae synthesize lipids from their available carbon source, which is both inorganic carbon such as carbon dioxide and organic carbon such as glucose. Depending on the carbon source used, the lipid content in microalgae can vary widely [52]. 

To evaluate the nutritional value of lipids in microalgae, the fatty acid distribution is crucial. The main groups of SFAs, MUFAs and PUFAs have substantial functions in the human body, e.g., energy storage and the modulation of cell signaling [53]. Meta-analyses associate an increased risk of cardiovascular diseases with an increased intake of SFAs due to their effect on inflammation processes and cholesterol metabolism, free fatty acids and triacylglycerols [54,55]. In this study, microalgae from the chromista kingdom had higher amounts of SFAs compared to the plantae microalgae. The SFA contents generally matched the literature data for *Microchloropsis*, *Nannochloropsis*, *Phaeodactylum* and *Tetraselmis* [56,57]. C16:0 is the most common SFA in the human body [58]. C16:0 can be consumed via diet or synthesized endogenously from other fatty acids, carbohydrates and amino acids. It is involved in several biological processes such as preserving the physical-chemical properties of membrane phospholipids and being the starting fatty acid for the elongation and desaturation of long-chain (LC) fatty acids [59]. An increased consumption of C16:0-rich foods is associated with an increased cardiovascular risk [60]. In *Nitzschia palea* (C16:0: 19% of total fatty acid methyl esters (FAME)) and *Tetradesmus obliquus* (C16:0: 17% of total FAME), lower C16:0 amounts were detected compared to the literature: 33% in *Nitzschia palea* and 22% in *Tetradesmus obliquus* [56,61]. An enormous variation in the C16:0 content was also observed in *Botryococcus braunii* (23% of total FAME) and *Klebsormidium* sp. (38% of total FAME) compared to 12 and 7%, respectively, in the literature [25,62]. In the present study, the microalgae from plantae had lower amounts of total SFA than chromista, which increases their nutritional value. The predominant MUFA in chromista microalgae was palmitoleic acid (C16:1_n7_), whereas oleic acid (C18:1_n9_) was the most dominant MUFA in plantae. In the literature, similar results were described for the fatty acid profiles of the species *Botryococcus*, *Eustigmatos*, *Phaeodactylum* and *Tetradesmus* [28,57,61,63]. C18:1_n9_ serves as a precursor for PUFAs as it can be metabolized by elongases and desaturases in the endoplasmic reticulum [64]. The Fe concentration in the culture medium affects the accumulation of C18 fatty acids, such as C18:0, C18:1_n7_ and C18:1_n9_, while the amounts of C16 fatty acids are not dependent [65]. Low nitrogen content also leads to higher contents of C18 fatty acids in microalgae [66]. LC n3-PUFA are well studied and maintain cardiovascular and mental health [67,68]. Due to the high contents of α-linoleic acid (ALA), EPA and DHA, 11 out of the 15 analyzed microalgae could be claimed 6 to be “high-omega-3 fatty acids” according to the European Commission Regulation (EC) 1924/200 [69]. The label “high-omega-3 fatty acids” is strictly reserved for at least 0.6 g ALA per 100 g and 100 kcal or at least 80 mg EPA+DHA per 100 g and per 100 kcal [69]. *A. lusatica*, *B. braunii*, *Stichococcus* sp. and *S. minor* provide more ALA than soy beans (1.6%), oats (1.4%) and olive oil (0.76%) [70]. The literature indicates higher ALA amounts for *Tetraselmis suecica* and *Tetradesmus obliquus* than the microalgae mentioned above [31,56]. The present data indicate that *P. tricornutum* and *M. salina*, as well as *Eustigmatos* sp., *N. limnetica* and *B. braunii,* contain more EPA than conventional EPA sources such as salmon (1.0%), herring (0.9%) and anchovy (0.8%) [71]. These findings are comparable with the data in the literature [28,56,57,72]. The cultivation phase had no effect on n3-PUFA concentration, which differs from previous available data. Teh et al. described an ALA decrease in *Chlorella vulgaris* during growth [73], while Fidalgo et al. determined an increase in ALA during the growth of *Isochrysis galbana* [74]. Cultivation conditions appear to affect the n3-PUFA amounts. While low nitrogen levels in the medium decrease n3-PUFA accumulation, higher salinity and phosphate amounts increase C18:3_n3_ content [63,73,75]. A balanced n6/n3 PUFA ratio of roughly 5:1 in the diet is suggested to prevent cardiovascular diseases [14]. The shift of the ratio to 10–20:1 in the Western diet may be related to increased obesity, low-density lipoprotein cholesterol concentrations and cardiovascular risk [76,77]. The average n6/n3 PUFA ratio of nearly 1:2 in chromista microalgae and 1:1 in plantae may be valuable for human health in terms of optimizing the n6/n3 PUFA ratio in the diet. Altogether, the fatty acid profiles of all microalgae varied substantially. The great differences between both kingdoms, apart from different cultivation factors, may be caused by the more complex membrane topology and, therefore, fatty acid composition in chromista [16]. Variations in the fatty acid profile between the growth and stationary phase as described in the literature were not seen, likely due to the small number of microalgae analyzed in the growth phase.

### 3.5. Pigments

Being crucial for photosynthesis and giving most microalgae their typical greenish color, chlorophyll is the most abundant natural pigment [78]. Microalgae can accumulate between 1 and 3% chlorophyll d.w. and, under perfect conditions, *Chlorella* sp. up to 5%, although commercially available *Chlorella* sp. powders are more likely to have 1–2% chlorophyll [1,79]. *A. lusatica* and *Klebsormidium* sp. seem to have higher chlorophyll concentration than commercially available microalgae [1]. While chlorophyll a is found in all microalgae, chlorophyll b is only present in *Chlorophyta* (plantae) and chlorophyll c only in microalgae from the chromista kingdom, such as *Haptophyta*, *Ochrophyta* and *Bacillariophyta* [80]. The amount of chlorophyll synthesized by microalga is changeable, and can be controlled by various factors such as the intensity and the wavelength of the light source [81]. Carotenoids are used as natural food coloring additives by the food industry but can also be valuable for human nutrition due to their antioxidative properties, as well as their role in the inhibition of tumor growth and induction of apoptosis [82,83,84]. Usually, most microalgae contain carotenoid amounts between 0.1 and 1.0%, which was in accordance with the analyzed microalgae [82,85,86]. It is well known from research that the amount of carotenoids in microalgae can be significantly increased under stress conditions such as nutrition deficiency, high UV exposure and salinity [87]. Furthermore, the carotenoid profile varies between microalgae. While fucoxanthin is the most present carotenoid in *P. tricornutum*, *T. suecica* is richer in lutein and violaxanthin and *Nannochloropsis gaditana* richer in violaxanthin and β-carotene [88]. Microalgae tend to increase their chlorophyll and carotenoid content during growth [81,89]. However, no differences were seen in the analyzed microalgae, which is most likely due to the low number of microalgae analyzed from different cultivation stages. 

### 3.6. Minerals and Trace Elements

Limited data regarding the micronutrient content in microalgae are available, since research and industry are more focused on nutrients, e.g., carotenoids and n3-PUFA. Because of the intrinsic composition of the cell walls and their negatively charged functional groups, microalgae are able to accumulate larger amounts of metals [90]. Microalgae in the stationary phase accumulated less minerals and trace elements compared to their counterparts in the growth phase. During the growth phase, microalgae perform more photosynthesis due to an increased energy requirement [91]. Fe plays a key role in the synthesis of chlorophyll, whereas Mg is bonded to the active center of chlorophyll [92,93]. Therefore, the higher accumulation of minerals and trace elements in the growth phase is plausible. Contrary results were obtained for Ni, Mo and I_2_. Stronger differences of both kingdoms were seen for Ca, As and Pb, which were higher in chromista, except for Pb. Previous studies showed the close link between minerals and trace element enrichment in microalgae. The Mn content in microalgae, for example, can be influenced by the amounts of Zn in the culture medium [90]. However, many synergistic interactions between elements are not fully understood and need further investigation. Ca is involved in many biological pathways and is essential for skeleton mineralization and bone health [94]. *P. tricornutum*, *C. carterae* and *T. suecica* are richer in Ca than common Ca-rich foods such as cabbage (212 mg/100 g) and milk (113 mg/100 mL) [95,96]. Nevertheless, quantities of 373 g (*P. tricornutum*), 387 g (*C. carterae*) and 59 g (*T. suecica*) need to be consumed to fulfill the recommended daily intake (RDI) of 1 g Ca from the German Nutrition Society [14]. Mg is used as cofactor in a variety of metabolic reactions in the human body such as protein and DNA synthesis [97]. Mg-rich microalgae such as *C. carterae*, *M. bisecta* and *T. suecica* could contribute to the optimal nutrient intake of this valuable mineral in human nutrition. Amounts of 28–33 g *M. bisecta,* the Mg-richest microalgae studied here, would cover the RDI of 300–350 mg Mg [14]. *M. bisecta* provides larger amounts of Fe than animal Fe sources such as pork liver (16 mg/100 g) and, therefore, may also help to reduce Fe deficiency in plant-based diets. The consumption of about 1 g *M. bisecta* could already fulfill the RDI for Fe (10–15 mg) [14]. *N. limnetica* may support the antioxidant and anti-inflammatory processes of selenoproteins due to its high content of Se compared to other Se sources such as fish (6–63 µg/100 g) and pork (27–35 µg/100 g) [98,99,100]. However, the necessary amounts to be consumed would be about 44 g to cover the RDI for selenium (60–70 µg) [14]. Zn is essential for wound healing, strengthening the immune system, cell differentiation and proliferation [101,102]. *C. carterae* might be a valuable source of Zn with similar amounts to other Zn sources such as cashew nuts (5.8 mg/100 g) and beef (5.0 mg/100 g) [103]. However, large quantities of 127–292 g *C. carterae* need to be consumed to cover the RDI of 7–16 mg Zn [14]. The consumable amounts of microalgae are often limited due to their distinctive smell, coloring of different foods and dryness. However, the addition of small amounts of microalgae biomass to the diet can help to achieve the RDI.

### 3.7. Heavy Metals

Heavy metal amounts in microalgae can be generally high due to bioaccumulation or biosorption [104]. Due to the negative impact of Hg, Cd and Pb on human health, their maximum tolerated concentration in microalgae is strictly regulated by the European Commission Regulation (EC) No.1881/2006. By inducing oxidative stress, heavy metals cause impaired neurobehavioral development in children, kidney damage, gastrointestinal diseases and carcinogenicity and affect the central nervous system [105]. Due to contamination of the central compressed air supply, the heavy metal levels were unusually high in the microalgae cultivated for this study and compared to cultivations of two of the species (*C. novae-angliae*, *M. salina*) on a technical scale. Maximum tolerable amounts of <100 µg/100 g for Cd were exceeded in every analyzed microalga except *N. palea* and *P. tricornutum*. Tolerable amounts of <10 µg/100 g for Hg were not exceeded in any microalga. The regulations for Pb with <300 µg/100 g microalgae were only fulfilled by *P. tricornutum, T. obliquus* and *M. salina* from the growth and stationary phase. Maximum contents of As are not yet regulated for microalgae powder. Additionally, a further classification of As is needed since organic As is far less toxic compared to inorganic arsenic species [106]. The accumulation of heavy metals showed a great variety between and within the same phyla as well as different and similar culture media, which is in accordance with the literature [107,108]. These data indicate that the accumulation of heavy metals does not only depend on the degree of heavy metal input during cultivation, which should, therefore, be carefully monitored and minimized. The amounts of heavy metals are also influenced by the functional groups (carboxyl, amino, hydroxyl and sulfate) of the cell wall. Microalgae with more functional groups in the outer layer of the cell wall tend to accumulate more heavy metals [109]. We assume that some microalgae such as *N. limnetica* and *T. suecica* might integrate more functional groups into their cell walls, which could have led to higher biosorption and bioaccumulation of heavy metals. However, the high concentrations of heavy metals were most likely caused by a contaminated gas supply. The compression air supply was located in the institute’s basement, sucking in air through an open, low-lying basement window near a crossroad. Further experiments analyzing the used air confirmed the contamination with heavy metals. Varying traffic density, weather and cultivation time as well as the use of different sterile filters at the air inlets of the cultivation bottles might have caused a difference in heavy metal contamination of the analyzed microalgae. Therefore, it is necessary to ensure a pure cultivation medium and gas supply for the production of microalgae for human nutrition.

### 3.8. Multi-Criteria Analysis of the Biomass Suitability for Nutrition

To determine the value of each microalgae for human nutrition, the microalgae were compared and ranked (Table 5). The amounts of SEA, EAA, protein, fiber, carotenoids, minerals, trace elements, n3-PUFA and a low n6/n3 ratio were determined as nutrients of high nutritional value. On the other hand, high amounts of SFAs as well as a high n6/n3 ratio lowered the nutritional value of the microalgae. The analyzed nutrients were weighted equally. We awarded plus points for essential or nutritionally favorable nutrients and minus points for negatively associated substances. Since the contamination conditions were not controlled and uniform, the heavy metal contents are not included in the weighting, but are listed. Further investigations of the species-specific tendency for heavy metal uptake are necessary. The toxicity of As differs in its organic and inorganic form. Since only the total amount of As was determined, no further statement about As toxicity can be made. Therefore, the As concentration was not included in the determination of the nutritional value of the microalgae. *C. novae-angliae* in the stationary phase had above average concentrations of I_2_ but was generally low in nutrients compared to the other analyzed microalgae. It was also characterized by a high n6/n3 ratio. This caused its lowest ranking for nutritional value. *Klebsormidium* sp. was rich in EAA, protein and Se but lacked in further nutrients and had the highest n6/n3 ratio in all analyzed microalgae, which resulted in it being ranked in 18th place. The 17th place was given to *T. suecica,* having valuable amounts of Mg, Ca, Se and I. The nutritional profile of *M. salina* in the stationary phase was defined by the highest content of SFAs but also valuable amounts of EPA and I_2_ as well as a low n6/n3 score, leading to its ranking in 16th place. The biomass of *C. novae-angliae* in the growth phase was rich in proteins with large amounts of SEA and EAA as well as fiber and Mg, yet the high n6/n3 ratio caused a ranking in 15th place. The 14th place was given to *S. minor* in the stationary phase with high contents of EAA, protein, fiber and I_2_ but also a slightly increased n6/n3 ratio. *N. limnetica* was ranked in 13th place. Regardless of its high amounts of EPA and trace elements, the nutritional value was reduced by the SFA concentrations. In 12th place is *T. obliquus* in the stationary phase, with valuable amounts of fiber and Ca and a low n6/n3 ratio but also increased concentrations of Hg. The highest ALA content and lowest n6/ratio followed by high amounts of EPA and fiber was determined in *B. braunii.* Nevertheless, its high SFA d concentration decreased its nutritional value, which led to a ranking in 11th place. Valuable amounts of various nutrients such as protein, Mg, Mn, Fe, Cu, Zn, Ni and Mo were determined in *N. palea.* Therefore, *N. palea* was ranked in 10th place among all microalgae. *Stichococcus* sp., in ninth place, was the third highest in EPA content and had valuable amounts of Mg, Se, Mo and I_2_ but also a slightly increased n6/n3 ratio. The second highest ALA and carotenoid content as well as high amounts of SEA, EAA, protein and Mg led to *A. lusatica* being ranked in eighth place. *Eustigmatos* sp. had a low n6/n3 ratio, was rich in EPA and fiber and was fourth for carotenoid content. The nutritional value was slightly decreased by its SFA amounts, which caused a ranking in seventh place. *S. minor* in the growth phase had the third highest fiber and protein content as well as a low n6/n3 ratio, ranking it the sixth most nutritional valuable microalgae. The second highest SEA, fourth highest fiber, third highest ALA concentrations and the third lowest n6/n3 ratio was determined in *T. obliquus* in the growth phase, ranking it the fifth most nutritional valuable among all the analyzed microalgae. *M. bisecta* was ranked in fourth place and defined by valuable amounts of protein and Zn as well as the highest concentrations of Mg, Mn, Fe, Cu, Ni and Mo, but also a high n6/n3 ratio among all the microalgae. With the highest amounts of carotenoids, the second highest EPA concentration, causing a low n6/n3 ratio, and valuable amounts of Mg, Ca, Cu and I_2_ but slightly increased SFA concentrations, *P. tricornutum* was ranked in third place. *M. salina* in the growth phase was the richest in protein and EPA and had the lowest n6/n3 ratio. Furthermore, it was rich in SEA, EAA, Mg, Ca, Cu and I. Therefore, it was ranked in second place. *C. carterae* was ranked as the most valuable microalgae for human nutrition. It was defined by the highest amounts of fiber, DHA, Zn, and I_2_ among all the microalgae. Furthermore, *C. carterae* was rich in EAA, carotenoids, Ca, Mg, Mn, Fe, Zn, Se, Ni and Mo and had a low n6/n3 ratio. It appears that due to their amounts of nutrients, *C. carterae* and *M. salina* might be more nutritionally valuable for human nutrition than microalgae such as *Arthrospira platensis*, *Dunaliella salina*, *Tetraselmis chuii* and some *Chlorella species*, which are already authorized for human nutrition by the European Commission [1].

## 4. Materials and Methods

### 4.1. Microalgae Biomass

Fifteen microalgae (7 to 12 g dry weight) from different kingdoms and harvest points were cultivated and provided by the Competence Center Algal Biotechnology of Anhalt University of Applied Sciences in Köthen, Germany, for the analysis of various nutrients (Table 6). The stationary phase was collected when the nitrogen source was drained, which was reached after about 21 days of growing. The growth phase was collected after about 7 days when nitrogen was still available from the cultivation medium. The biomass was washed, centrifuged, ground with a ball mill, freeze-dried and stored at −80 °C until nutrient analysis. Information regarding the cultivation of the microalgae such as natural habitat, culture medium, photobioreactor and point of harvest, as well as further classification, are listed in Table 6. Contamination of the provided microalgae biomass with microorganisms and other species was kept to a minimum and the samples were examined constantly via microscopy.

### 4.2. Amino Acid and Ammonium Quantification

Amino acids were obtained through the hydrolysis of lyophilized microalgae biomass and quantified using ion exchange chromatography with post column ninhydrin derivatization. A total of 20 mg dry algae biomass was subjected to acidic hydrolysis (5 mL 6 N HCl, 48 h, 110 °C, nitrogen as protective gas). Similarly, another aliquot of 20 mg dry algae biomass was subjected to alkaline hydrolysis (5 mL 4 M NaOH, 24 h, 110 °C, nitrogen as protective gas) for the determination of tryptophan, which is unstable otherwise. The alkaline hydrolysate was acidified with HCl to pH < 2. Both acidic and acidified hydrolysate were evaporated, and the residues were equilibrated in 5 mL sodium citrate buffer pH 2.2. Amino acid analysis was carried out on a Biochrom30+ analyzer (Biochrom, Cambridge, UK) via separation on a cation exchange resin, post column ninhydrin derivatization and detection at 440 and 570 nm, according to the manufacturer’s instructions. Norleucine was added as an internal standard. Buffers and standards were purchased from Laborservice Onken (Laborservice Onken, Gruendau, Germany). Based upon duplicate analyses, the accuracy of the obtained individual amino acid and ammonium contents was, on average, ±15% (RSD) including low abundant amino acids. 

### 4.3. N-Factor Calculation

The calculation of the N-factor was performed with the concentration of amino acids, nitrogen-containing molecules and the total organic N content as described by Sandgruber et al. [1]. Amino acids with concentrations under 0.001 g/100 g were set to 0.001 g/100 g for the calculation of the N-factor.

### 4.4. Macronutrients

The total nitrogen and protein nitrogen content was analyzed with the Kjeldahl method according to DIN EN ISO 14891 2002-07 and Matissek et al. [110]. Pure (total nitrogen) and crude protein content (protein nitrogen) were calculated by multiplying the nitrogen content with a general constant (6.25) or a specific N-factor for microalgae found in the literature (4.97) or determined as mentioned above [1,36]. With the Total Dietary Fiber Kit BIOQUANT^®^ (Merck, Darmstadt, Germany), the content of total fiber was enzymatically determined [111]. Total fat content was analyzed with a combination of Weibull–Stoldt hydrolysis and Soxhlet extraction according to ASU L 06.00-6. The microalgae were hydrolyzed with HCl following the extraction of fat with petroleum ether.

### 4.5. Fatty Acid Analysis

For the analysis of the fatty acid profile, the lipids were extracted with a modified Folch/Bligh and Dyer method [112]. The lipids were saponified and methylated with sodium methoxide and boron trifluoride. Afterwards, the resulting FAMEs were isolated and analyzed via gas chromatography (GC; GC-17 V3, Shimadzu, Duisburg, Germany) equipped with an AOC-5000 auto-sampler and flame ionization detector. H_2_ was used as the carrier gas and the column was a fused-silica capillary DB-225ms column (30 m × 0.25 mm, i.d. with 0.1 μm film thickness; J and W Scientific, Folsom, CA, USA). The quantification of FAMEs was performed with GC solution software (LabSolution LC/GC release 5.92, Shimadzu, Kyoto, Japan).

### 4.6. Total Carotenoids and Chlorophylls

Pigments were extracted from lyophilized biomass and quantified spectrophotometrically. A total of 10 mg dry algae biomass was dispersed in 0.25 mL ice-cold aqueous acetone (90%) and disintegrated with 0.5 mL glass grinding beads (ø 0.75-1 mm) in a vibrating mill (MM 400, Retsch, Haan, Germany) at 30 Hz for 20 min. Another 0.75 mL ice-cold aqueous acetone (90%) was added after bead beating, and the extract was separated through centrifugation and collected. The extraction step was repeated until the biomass pellet was decolorized. The extracts were combined and made up to 10 mL in volumetric flasks. The absorbance of the appropriately diluted extracts was measured at 450, 630, 647, 664 and 750 nm (Specord 50 Plus, Analytik Jena, Jena, Germany) in quartz cuvettes with a 10 mm pathlength. Equations of Jeffrey and Humphrey were used to calculate chlorophyll a, chlorophyll b and chlorophyll c1 + c2 [113]. Total carotenoid content was calculated according to the method of Jaspers [114], although it must be noted that this equation is not suited for fucoxanthin-containing species from the bacillariophyta and haptophyta due to the low specific absorption coefficient of fucoxanthin at 450 nm, as compared with other carotenoids, and due to chlorophyll c interference [115]. 

### 4.7. Element Analysis (CHS)

Simultaneous CHS analysis was carried out on a vario MICRO cube analyzer (Elementar Analysensysteme, Hanau, Germany). A total of 2–4 mg dry algae biomass was wrapped in tin foil and subjected to high-temperature combustion (1150 °C) in an oxygen-rich atmosphere. After purification and reduction (850 °C), the gas mixture (CO_2_, H_2_O, N_2_, SO_2_) was separated on adsorption columns and then detected by means of a thermal conductivity detector. Sulfanilamide served as the reference substance. Based upon triplicate analyses, the accuracy was, on average, ±2% (RSD) for C and H and ±8% (RSD) for S.

### 4.8. Minerals, Trace Elements and Heavy Metals

The microalgae were digested in a microwave pressure digestion system and a 10% HNO_3_ solution with 1 μg/L Rh. For the multi-element detection, inductively coupled plasma mass spectrometry with tandem spectrometry (Agilent ICP-QQQ-MS 8800, Agilent Technologies, Waldbronn, Germany) was performed. In mass-shift mode, a reaction/collision gas mixture of oxygen and hydrogen was used. To avoid interference, helium was used as the collision gas in on-mass mode. Furthermore, a 10 μg/L solution with ^77^Se, a 10 μg/L solution with naturally occurring Se and a mixture by halves of ^77^Se and natural Se were used for the Se isotope dilution analysis. Daily tuning of the nebulizer gas flow, parameters of lenses, Q1, collision cell and Q2 was performed to guarantee the maximum sensitivity of the analysis (oxide ratio < 1.0 % (^140^Ce^16^O^+^/^140^Ce^+^), doubly charged ratio < 1.5 % (^140^Ce^2+^/^140^Ce^+^), background counts < 0.1 cps). Every 25 samples, the calibration blanks and recalibration checkpoints were newly analyzed.

### 4.9. Statistical Analysis

The measured parameters are expressed as means with standard deviation. Statistical analysis was performed with SPSS Statistics version 27 Premium. Normal distribution was detected with the Shapiro–Wilk test. Statistical significance between the different kingdoms of the analyzed microalgae was detected at normal distribution via the unpaired t-test in combination with the Levene test for identifying the homogeneity of variances. In cases without normal distribution, the Mann–Whitney test was performed. To calculate statistically significant differences between both cultivation phases, the paired t-test was performed with normal distribution; otherwise, the Wilcoxon test was used. When concentrations showed values below the LOQ, the LOQ was used for calculating statistical significance. If more than 50% of the data of one parameter were under the LOQ, no statistical analysis was performed. *p*-values < 0.05 were defined as statistically significant.

## 5. Conclusions

Overall, the nutrient profile of all the analyzed microalgae showed great diversity. The species of the microalgae, its cultivation phase and various factors during its cultivation influence the nutritional profile and value for human nutrition. The amino acid profile and content of nitrogen compounds from both kingdoms was not significantly different. Furthermore, the total carotenoid and chlorophyll content between different kingdoms and cultivation phases was similar. Comparing the kingdoms, higher contents of total fat, C14:0, C16:1_n7_, C20:4_n6_, C20:5_n3_ and As were detected in chromista, while higher contents of C20:0, C20:1_n9_, C18:3_n3_, Ca and Pb emerged in plantae. During the stationary phase, the accumulation of Ni, Mo and I_2_ was higher compared to the growth phase. The contents of arginine, histidine, ornithine, pure and crude protein, Mg, Mn, Fe, Zn and As were higher in microalgae in the growth phase. Further differences between cultivation phases might have failed to be indicated due the small number of analyzed microalgae. With high levels of I_2_ and moderate levels of EAA, SEA, protein, fiber, n3-PUFA, carotenoids, minerals and trace elements but a high n6/n3 ratio, *C. novae-angliae* in the stationary phase had the least nutritional value. The highest value for human nutrition was determined for *C. carterae. C. carterae* was rich in fibers, carotenoids, C20:6_n3_, Mg, Ca, Mn, Fe, Zn, Se, Ni, Mo and I_2_ and had a low n6/n3 ratio. Furthermore, the concentrations of heavy metals and SFAs was low compared to the other analyzed microalgae. The consideration of heavy metals in the evaluation of the nutritional value of some microalgae such as *M. salina*, *N. palea* or *M. bisecta* might decrease their value slightly. The amount of microalgae to be consumed to cover the RDI of various nutrients is often too large. Thus, specific microalgae will not serve as exclusive macro- and micronutrient sources but as a complement. However, microalgae can make an important contribution to meeting the need for various nutrients for human nutrition.

## Figures and Tables

**Table 5:** Nutritional ranking of all analyzed microalgae.

Ranking	Name	Kingdom	CP	Positive Characteristics	Negative Characteristics
1.	*Chrysotila carterae*	Cr	SP	↑EAA, ↑↑↑Fiber, ↑↑Carotenoids, ↑↑↑DHA, ↓↓n6/n3, ↑↑Mg, ↑↑↑Ca, ↑Mn, ↑↑Fe, ↑↑↑Zn, ↑Se, ↑↑Ni, ↑↑Mo, ↑↑↑I_2_	(↑↑As)
2.	*Microchloropsis salina*	Cr	GP	↑↑SEA, ↑↑↑EAA, ↑↑↑ Protein, ↑↑↑EPA, ↓↓↓n6/n3, ↑Mg, ↑Ca, ↑↑Zn, ↑I_2_	(↑Hg), (↑↑↑As)
3.	*Phaeodactylum tricornutum*	Cr	SP	↑↑↑EPA, ↓↓n6/n3, ↑↑↑Carotenoids, ↑Mg, ↑↑Ca, ↑↑Cu, ↑I_2_	↑SFA, (↑↑As)
4.	*Myrmecia bisecta*	Pl	SP	↑↑Protein, ↑↑↑Mg, ↑↑↑Mn, ↑↑↑Fe, ↑↑↑Cu, ↑Zn, ↑↑↑Ni, ↑↑↑Mo	↑↑n6/n3,(↑↑Pb, ↑↑Hg) (↑↑As)
5.	*Tetradesmus obliquus*	Pl	GP	↑↑↑SEA, ↑↑Fiber, ↑↑ALA, ↓↓n6/n3	
6.	*Spongiochloris minor*	Pl	GP	↑↑Protein, ↑↑↑Fiber, ↓n6/n3	
7.	*Eustigmatos* sp.	Cr	SP	↑↑Fiber, ↑EPA, ↓n6/n3, ↑↑Carotenoids	↑SFA
8.	*Autumnella lusatica*	Pl	SP	↑SEA, ↑EAA, ↑↑Protein, ↑↑ALA, ↑↑↑Carotenoids, ↑Mg	(↑↑Pb)
9.	*Stichococcus* sp.	Pl	SP	↑↑EPA, ↑Mg, ↑Se, ↑Mo, ↑I_2_	↑n6/n3,
10.	*Nitzschia palea*	Cr	SP	↑↑Protein,↑Mg, ↑Mn, ↑↑Fe, ↑↑Cu, ↑↑Zn, ↑Ni, ↑↑Mo,	(↑↑↑Hg)
11.	*Botryococcus braunii*	Pl	SP	↑↑Fiber, ↑↑↑ALA, ↑EPA, ↑↑↑n6/n3	↑↑SFA, (↑↑Cd)
12.	*Tetradesmus obliquus*	Pl	SP	↑↑Fiber, ↓n6/n3, ↑↑Ca	(↑↑Hg)
13.	*Nannochloropsis limnetica*	Cr	SP	↑↑EPA, ↑↑↑Mn, ↑↑Fe, ↑↑↑Se, ↑Ni, ↑Mo	↑↑SFA, (↑↑↑Cd, ↑↑↑Pb)
14.	*Spongiochloris minor*	Pl	SP	↑↑EAA, ↑↑Protein, ↑↑↑Fiber, ↑I_2_	↑n6/n3, (↑Hg)
15.	*Chlorococcum novae-angliae*	Pl	GP	↑↑SEA, ↑↑↑EAA, ↑↑↑Protein, ↑↑Fiber, ↑Mg	↑↑n6/n3, (↑↑Hg), (↑↑ Pb)
16.	*Microchloropsis salina*	Cr	SP	↑↑EPA, ↓n6/n3, ↑I_2_	↑↑↑SFA, (↑↑↑As)
17.	*Tetraselmis suecica*	Pl	SP	↑↑Mg, ↑↑↑Ca, ↑Se, ↑↑I_2_	(↑↑Cd, ↑↑↑Pb)
18.	*Klebsormidium* sp.	Pl	SP	↑↑EAA, ↑↑Protein, ↑Se	↑↑↑n6/n3, (↑↑Cd)
19.	*Chlorococcum novae-angliae*	Pl	SP	↑I_2_	↑↑n6/n3,(↑↑Hg, ↑↑Pb)

ALA, alpha-linolenic acid; CP, cultivation phase; Cr, chromista; EAA, essential amino acids; EPA, eicosapentaenoic acid; DHA, docosahexaenoic acid; GP, growth phase; Pl, plantae; SEA, semi-essential amino acids; SFA, saturated fatty acids; SP, stationary phase.

**Table 6:** Classification and cultivation information of 15 microalgae species.

Microalgae (Strain Number)	Kingdom	Phylum	Class	Habitat	Culture Medium	PBR	Point of Harvest
*Chrysotila carterae* (SAG 944-1)	*Cr*	*Haptophyta*	*Coccolithophyceae*	Marine	SWES	CF	SP
*Eustigmatos* sp. (KASC I-005)	*Cr*	*Ochrophyta*	*Eustigmatophyceae*	Aeroterrestrial	BBM	BC	SP
*Microchloropsis salina* (SAG 40.85)	*Cr*	*Ochrophyta*	*Eustigmatophyceae*	Marine	f/2	BC	GP, SP
*Nannochloropsis* *limnetica* (SAG 18.99)	*Cr*	*Ochrophyta*	*Eustigmatophyceae*	Fresh water	OHM	BC	SP
*Nitzschia palea* (KASC I-007)	*Cr*	*Bacillariophyta*	*Bacillariophyceae*	Fresh water	BBM +Na_2_SiO_3_	CF	SP
*Phaeodactylum* *tricornutum* (SAG 1090-1b)	*Cr*	*Bacillariophyta*	*Bacillariophyta* *classis incertae sedis*	Marine	1/2 SWES	CF	SP
*Autumnella lusatica* (Hindak 2012/2)	*Pl*	*Chlorophyta*	*Trebouxiophyceae*	Fresh water	KUHL	BC	SP
*Botryococcus braunii* (University of Tokyo S. Okada)	*Pl*	*Chlorophyta*	*Trebouxiophyceae*	Fresh water	BG11	BC	SP
*Chlorococcum* *novae-angliae* (SAG 5.85)	*Pl*	*Chlorophyta*	*Chlorophyceae*	Fresh water	ES	CF	GP, SP
*Klebsormidium* sp. (KASC I-008)	*Pl*	*Charophyta*	*Klebsormidiophyceae*	Aeroterrestrial	BBM/Šetlik (1:1)	BC	SP
*Myrmecia bisecta* (SAG 2043)	*Pl*	*Chlorophyta*	*Trebouxiophyceae*	Terrestrial	BBM	BC	SP
*Spongiochloris minor* (KASC 29.01)	*Pl*	*Chlorophyta*	*Chlorophyceae*	Terrestrial	BBM	CF	GP, SP
*Stichococcus* sp. (KASC I-30-01)	*Pl*	*Chlorophyta*	*Trebouxiophyceae*	Aeroterrestrial	BBM/Šetlik (2:1)	BC	SP
*Tetradesmus obliquus* (SAG 276-1)	*Pl*	*Chlorophyta*	*Chlorophyceae*	Fresh water	BBM	CF	GP, SP
*Tetraselmis suecica* (CCAP 66/38)	*Pl*	*Chlorophyta*	*Chlorodendrophyceae*	Marine	SWES	BC	SP

BC, bubble column; Cr, chromista; CF, cultivation flask; Pl, plantae; PBR, photobioreactor; GP, growth phase; SP, stationary phase.

## Data Availability

The data that support the findings of this study are available from the corresponding authors, [C.D.; C.G.], upon reasonable request.

## References

[1] Sandgruber F., Gielsdorf A., Baur A.C., Schenz B., Muller S.M., Schwerdtle T., Stangl G.I., Griehl C., Lorkowski S., Dawczynski C. (2021). Variability in macro- and micronutrients of 15 commercially available microalgae powders. Mar. Drugs.

[2] European Commission (2017). Commission Implementing Regulation (EU) 2017/2470 of 20 December 2017 Establishing the Union List of Novel Foods in Accordance with Regulation (EU) 2015/2283 of the European Parliament and of the Council on Novel Foods.

[3] Pierre G., Delattre C., Dubessay P., Jubeau S., Vialleix C., Cadoret J.-P., Probert I., Michaud P. (2019). What is in store for eps microalgae in the next decade?. Molecules.

[4] Richmond A. (2004). Handbook of Microalgal Culture: Biotechnology and Applied Phycology.

[5] You J., Mallery K., Mashek D.G., Sanders M., Hong J., Hondzo M. (2020). Microalgal swimming signatures and neutral lipids production across growth phases. Biotechnol. Bioeng..

[6] Sui Y., Muys M., Vermeir P., D’Adamo S., Vlaeminck S.E. (2019). Light regime and growth phase affect the microalgal production of protein quantity and quality with dunaliella salina. Bioresour. Technol..

[7] Lourenço S.O., Barbarino E., Marquez U.M.L., Aidar E. (1998). Distribution of intracellular nitrogen in marine microalgae: Basis for the calculation of specific nitrogen-to-protein conversion factors. J. Phycol..

[8] Choudhary A., Karmakar R., Kundu K., Dahake V. (2011). “Algal” biodiesel: Future prospects and problems. Water Eenrgy Int..

[9] Hodgson P.A., Henderson R.J., Sargent J.R., Leftley J.W. (1991). Patterns of variation in the lipid class and fatty acid composition of nannochloropsis oculata (eustigmatophyceae) during batch culture. J. Appl. Phycol..

[10] Schwarzhans J.-P., Cholewa D., Grimm P., Beshay U., Risse J.-M., Friehs K., Flaschel E. (2015). Dependency of the fatty acid composition of euglena gracilis on growth phase and culture conditions. J. Appl. Phycol..

[11] Tossavainen M., Ilyass U., Ollilainen V., Valkonen K., Ojala A., Romantschuk M. (2019). Influence of long term nitrogen limitation on lipid, protein and pigment production of euglena gracilis in photoheterotrophic cultures. PeerJ.

[12] Boussiba S., Bing W., Yuan J.-P., Zarka A., Chen F. (1999). Changes in pigments profile in the green alga haeamtococcus pluvialis exposed to environmental stresses. Biotechnol. Lett..

[13] Niestroj I. (2000). Praxis der Orthomolekularen Medizin: Physiologische Grundlagen. Therapie mit Mikronährstoffen.

[14] Deutsche Gesellschaft für Ernährung (DGE), Österreichische Gesellschaft für Ernährung (ÖGE), Schweizerische Gesellschaft für Ernährung (SGE) (2021). Referenzwerte für die Nährstoffzufuhr.

[15] European Commission (2006). Commission Regulation (EC) No 1881/2006 of 19 December 2006 Setting Maximum Levels for Certain Contaminants in Foodstuffs.

[16] Cavalier-Smith T. (2018). Kingdom chromista and its eight phyla: A new synthesis emphasising periplastid protein targeting, cytoskeletal and periplastid evolution, and ancient divergences. Protoplasma.

[17] Cavalier-Smith T. (1999). Principles of protein and lipid targeting in secondary symbiogenesis: Euglenoid, dinoflagellate, and sporozoan plastid origins and the eukaryote family tree 1, 2. J. Eukaryot. Microbiol..

[18] Sahoo D., Seckbach J. (2015). The Algae World.

[19] Mišurcová L., Buňka F., Vávra Ambrožová J., Machů L., Samek D., Kráčmar S. (2014). Amino acid composition of algal products and its contribution to rdi. Food Chem..

[20] Tibbetts S.M., Milley J.E., Lall S.P. (2015). Chemical composition and nutritional properties of freshwater and marine microalgal biomass cultured in photobioreactors. J. Appl. Phycol..

[21] Cai Y., Zhai L., Fang X., Wu K., Liu Y., Cui X., Wang Y., Yu Z., Ruan R., Liu T. (2022). Effects of c/n ratio on the growth and protein accumulation of heterotrophic chlorella in broken rice hydrolysate. Biotechnol. Biofuels Bioprod..

[22] Godfray H.C.J., Beddington J.R., Crute I.R., Haddad L., Lawrence D., Muir J.F., Pretty J., Robinson S., Thomas S.M., Toulmin C. (2010). Food security: The challenge of feeding 9 billion people. Science.

[23] Nosworthy M.G., Franczyk A.J., Medina G., Neufeld J., Appah P., Utioh A., Frohlich P., House J.D. (2017). Effect of processing on the in vitro and in vivo protein quality of yellow and green split peas (*Pisum sativum*). J. Agric. Food Chem..

[24] Matissek R., Baltes W. (2015). Lebensmittelchemie.

[25] Fang H., Zhuang Z., Huang L., Zhao W., Niu J.J.F.I.N. (2022). *Dietary klebsormidium* sp. Supplementation improves growth performance, antioxidant and anti-inflammatory status, metabolism, and mid-intestine morphology of litopenaeus vannamei. Front. Nutr..

[26] Brown M.R. (1991). The amino-acid and sugar composition of 16 species of microalgae used in mariculture. J. Exp. Mar. Biol. Ecol..

[27] Miao G., Zhu C., Wang J., Tan Z., Wang L., Liu J., Kong L., Sun Y. (2015). Efficient one-pot production of 1,2-propanediol and ethylene glycol from microalgae (*Chlorococcum* sp.) in water. Green Chem..

[28] Ramaraj R., Kawaree R., Unpaprom Y. (2016). Direct transesterification of microalga botryococcus braunii biomass for biodiesel production. Emergent Life Sci. Res..

[29] Teuling E., Wierenga P.A., Schrama J.W., Gruppen H. (2017). Comparison of protein extracts from various unicellular green sources. J. Agric. Food Chem..

[30] Pollio A., Aliotta G., Pinto G., Paterno M., Bevilacqua A. (1997). Ecophysiological characters and biochemical composition of stichococcus bacillaris naegeli strains from low ph environments. Algol. Stud./Arch. Hydrobiol..

[31] Oliveira C.Y.B., Oliveira C.D., Prasad R., Ong H.C., Araujo E., Nisha S., Galvez A. (2021). A multidisciplinary review of tetradesmus obliquus: A microalga suitable for large-scale biomass production and emerging environmental applications. Rev. Aquac..

[32] Psachoulia P., Chatzidoukas C. (2021). Illumination policies for *Stichococcus* sp. Cultures in an optimally operating lab-scale pbr toward the directed photosynthetic production of desired products. Sustainability.

[33] Abid A.L., Bchir F., Hamdi M. (2017). Feasibility of carbon dioxide sequestration by *Spongiochloris* sp. microalgae during petroleum wastewater treatment in airlift bioreactor. Bioresour. Technol..

[34] Andreeva A.P., Budenkova E., Babich O., Sukhikh S., Ulrikh E., Ivanova S., Prosekov A., Dolganyuk V. (2021). Production, purification, and study of the amino acid composition of microalgae proteins. Molecules.

[35] Wild K.J., Steingass H., Rodehutscord M. (2018). Variability in nutrient composition and in vitro crude protein digestibility of 16 microalgae products. J. Anim. Physiol. Anim. Nutr..

[36] Lourenco S.O., Barbarino E., Lavin P.L., Marque U.M.L., Aidar E. (2004). Distribution of intracellular nitrogen in marine microalgae: Calculation of new nitrogen-to-protein conversion factors. Eur. J. Phycol..

[37] González López C.V., García M.D.C.C., Fernández F.G.A., Bustos C.S., Chisti Y., Sevilla J.M.F. (2010). Protein measurements of microalgal and cyanobacterial biomass. Bioresour. Technol..

[38] Metsoviti M.N., Katsoulas N., Karapanagiotidis I.T., Papapolymerou G. (2019). Effect of nitrogen concentration, two-stage and prolonged cultivation on growth rate, lipid and protein content of chlorella vulgaris. J. Chem. Technol. Biotechnol..

[39] Da Costa F., Le Grand F., Quéré C., Bougaran G., Cadoret J.P., Robert R., Soudant P. (2017). Effects of growth phase and nitrogen limitation on biochemical composition of two strains of tisochrysis lutea. Algal Res..

[40] Soliman G.A. (2019). Dietary fiber, atherosclerosis, and cardiovascular disease. Nutrients.

[41] Patel A.K., Singhania R.R., Awasthi M.K., Varjani S., Bhatia S.K., Tsai M.-L., Hsieh S.-L., Chen C.-W., Dong C.-D. (2021). Emerging prospects of macro- and microalgae as prebiotic. Microb. Cell Factories.

[42] Dhingra D., Michael M., Rajput, Patil R. (2012). Dietary fibre in foods: A review. J. Food Sci. Technol..

[43] Molino A., Iovine A., Casella P., Mehariya S., Chianese S., Cerbone A., Rimauro J., Musmarra D. (2018). Microalgae characterization for consolidated and new application in human food, animal feed and nutraceuticals. Int. J. Environ. Res. Public Health.

[44] Germán-Báez L.J., Valdez-Flores M.A., Félix-Medina J.V., Norzagaray-Valenzuela C.D., Santos-Ballardo D.U., Reyes-Moreno C., Shelton L.M., Valdez-Ortiz Á. (2017). Chemical composition and physicochemical properties of phaeodactylum tricornutum microalgal residual biomass. Food Sci. Technol. Int..

[45] Zhuang L.-L., Azimi Y., Yu D., Wang W.-L., Wu Y.-H., Dao G.-H., Hu H.-Y. (2016). Enhanced attached growth of microalgae scenedesmus. Lx1 through ambient bacterial pre-coating of cotton fiber carriers. Bioresour. Technol..

[46] Kent M., Welladsen H.M., Mangott A., Li Y. (2015). Nutritional evaluation of australian microalgae as potential human health supplements. PLoS ONE.

[47] Mutaf T., Oz Y., Kose A., Elibol M., Oncel S.S. (2019). The effect of medium and light wavelength towards stichococcus bacillaris fatty acid production and composition. Bioresour. Technol..

[48] Schädler T., Caballero Cerbon D., de Oliveira L., Garbe D., Brück T., Weuster-Botz D. (2019). Production of lipids with microchloropsis salina in open thin-layer cascade photobioreactors. Bioresour. Technol..

[49] Ma Y., Wang Z., Yu C., Yin Y., Zhou G. (2014). Evaluation of the potential of 9 nannochloropsis strains for biodiesel production. Bioresour. Technol..

[50] Zhou W., Wang H., Zheng L., Cheng W., Gao L., Liu T. (2019). Comparison of lipid and palmitoleic acid induction of tribonema minus under heterotrophic and phototrophic regimes by using high-density fermented seeds. Int. J. Mol. Sci..

[51] Wang H., Zhang Y., Zhou W., Noppol L., Liu T. (2018). Mechanism and enhancement of lipid accumulation in filamentous oleaginous microalgae tribonema minus under heterotrophic condition. Biotechnol. Biofuels.

[52] Ma X., Mi Y., Zhao C., Wei Q. (2022). A comprehensive review on carbon source effect of microalgae lipid accumulation for biofuel production. Sci. Total Environ..

[53] de Carvalho C.C.C.R., Caramujo M.J. (2018). The various roles of fatty acids. Molecules.

[54] Zhu Y., Bo Y., Liu Y. (2019). Dietary total fat, fatty acids intake, and risk of cardiovascular disease: A dose-response meta-analysis of cohort studies. Lipids Health Dis..

[55] Calder P.C. (2015). Functional roles of fatty acids and their effects on human health. J. Parenter. Enter. Nutr..

[56] Viso A.-C., Marty J.-C. (1993). Fatty acids from 28 marine microalgae. Phytochemistry.

[57] Krzemińska I., Nosalewicz A., Reszczyńska E., Pawlik-Skowrońska B. (2020). Enhanced light-induced biosynthesis of fatty acids suitable for biodiesel production by the yellow-green alga eustigmatos magnus. Energies.

[58] Carta G., Murru E., Banni S., Manca C. (2017). Palmitic acid: Physiological role, metabolism and nutritional implications. Front. Physiol..

[59] Carta G., Murru E., Lisai S., Sirigu A., Piras A., Collu M., Batetta B., Gambelli L., Banni S. (2015). Dietary triacylglycerols with palmitic acid in the sn-2 position modulate levels of n-acylethanolamides in rat tissues. PLoS ONE.

[60] Korbecki J., Bajdak-Rusinek K. (2019). The effect of palmitic acid on inflammatory response in macrophages: An overview of molecular mechanisms. Inflamm. Res..

[61] Mahmoud E.A., Farahat L.A., Abdel Aziz Z.K., Fatthallah N.A., Salah El Din R.A. (2015). Evaluation of the potential for some isolated microalgae to produce biodiesel. Egypt. J. Pet..

[62] Dilia P., Kalsum L., Rusdianasari R. (2018). Fatty acids from microalgae botryococcus braunii for raw material of biodiesel. J. Phys. Conf. Ser..

[63] Shen P.-L., Wang H.-T., Pan Y.-F., Meng Y.-Y., Wu P.-C., Xue S. (2016). Identification of characteristic fatty acids to quantify triacylglycerols in microalgae. Front. Plant Sci..

[64] Fernandes T., Fernandes I., Andrade C.A.P., Cordeiro N. (2016). Changes in fatty acid biosynthesis in marine microalgae as a response to medium nutrient availability. Algal Res..

[65] Lin Q., Gu N., Lin J. (2012). Effect of ferric ion on nitrogen consumption, biomass and oil accumulation of a scenedesmus rubescens-like microalga. Bioresour. Technol..

[66] Tan Y., Lin J. (2011). Biomass production and fatty acid profile of a scenedesmus rubescens-like microalga. Bioresour. Technol..

[67] Innes J.K., Calder P.C. (2020). Marine omega-3 (n-3) fatty acids for cardiovascular health: An update for 2020. Int. J. Mol. Sci..

[68] Grosso G., Galvano F., Marventano S., Malaguarnera M., Bucolo C., Drago F., Caraci F. (2014). Omega-3 fatty acids and depression: Scientific evidence and biological mechanisms. Oxid. Med. Cell. Longev..

[69] Lähteenmäki-Uutela A., Rahikainen M., Camarena-Gómez M.T., Piiparinen J., Spilling K., Yang B. (2021). European union legislation on macroalgae products. Aquac. Int..

[70] Rodriguez-Leyva D., Dupasquier C.M., McCullough R., Pierce G.N. (2010). The cardiovascular effects of flaxseed and its omega-3 fatty acid, alpha-linolenic acid. Can. J. Cardiol..

[71] Uauy R., Valenzuela A. (1992). Marine oils as a source of omega-3 fatty acids in the diet: How to optimize the health benefits. Prog. Food Nutr. Sci..

[72] Mohammady N., Maghraby D., Ibrahim E. (2014). Growth and oil production of nannochloropsis salina cultivated under multiple stressors. J. Pure Appl. Microbiol..

[73] Teh K.Y., Loh S.H., Aziz A., Takahashi K., Effendy A.W.M., Cha T.S. (2021). Lipid accumulation patterns and role of different fatty acid types towards mitigating salinity fluctuations in chlorella vulgaris. Sci. Rep..

[74] Fidalgo J.P., Cid A., Torres E., Sukenik A., Herrero C. (1998). Effects of nitrogen source and growth phase on proximate biochemical composition, lipid classes and fatty acid profile of the marine microalga isochrysis galbana. Aquaculture.

[75] Anne-Marie K., Yee W., Loh S.H., Aziz A., Cha T.S. (2020). Effects of excess and limited phosphate on biomass, lipid and fatty acid contents and the expression of four fatty acid desaturase genes in the tropical selenastraceaen messastrum gracile se-mc4. Appl. Biochem. Biotechnol..

[76] Wijendran V., Hayes K.C. (2004). Dietary n-6 and n-3 fatty acid balance and cardiovascular health. Annu. Rev. Nutr..

[77] Simopoulos A.P. (2000). Human requirement for n-3 polyunsaturated fatty acids. Poult. Sci..

[78] da Silva J.C., Lombardi A.T., Jacob-Lopes E., Queiroz M.I., Zepka L.Q. (2020). Chlorophylls in microalgae: Occurrence, distribution, and biosynthesis. Pigments from Microalgae Handbook.

[79] Marks G.S. (1966). The biosynthesis of heme and chlorophyll. Bot. Rev..

[80] Stengel D.B., Connan S., Popper Z.A. (2011). Algal chemodiversity and bioactivity: Sources of natural variability and implications for commercial application. Biotechnol. Adv..

[81] Gatamaneni Loganathan B., Orsat V., Lefsrud M., Wu B.S. (2020). A comprehensive study on the effect of light quality imparted by light-emitting diodes (leds) on the physiological and biochemical properties of the microalgal consortia of chlorella variabilis and scenedesmus obliquus cultivated in dairy wastewater. Bioprocess Biosyst. Eng..

[82] Ambati R.R., Gogisetty D., Aswathanarayana R.G., Ravi S., Bikkina P.N., Bo L., Yuepeng S. (2018). Industrial potential of carotenoid pigments from microalgae: Current trends and future prospects. Crit. Rev. Food Sci. Nutr..

[83] Pérez-Gálvez A., Viera I., Roca M. (2020). Carotenoids and chlorophylls as antioxidants. Antioxidants.

[84] Milani A., Basirnejad M., Shahbazi S., Bolhassani A. (2017). Carotenoids: Biochemistry, pharmacology and treatment. Br. J. Pharmacol..

[85] Ahmed F., Fanning K., Netzel M., Turner W., Li Y., Schenk P.M. (2014). Profiling of carotenoids and antioxidant capacity of microalgae from subtropical coastal and brackish waters. Food Chem..

[86] Li Z., Sun M., Li Q., Li A., Zhang C. (2012). Profiling of carotenoids in six microalgae (eustigmatophyceae) and assessment of their β-carotene productions in bubble column photobioreactor. Biotechnol. Lett..

[87] Coesel S.N., Baumgartner A.C., Teles L.M., Ramos A.A., Henriques N.M., Cancela L., Varela J.C.S. (2008). Nutrient limitation is the main regulatory factor for carotenoid accumulation and for psy and pds steady state transcript levels in dunaliella salina (chlorophyta) exposed to high light and salt stress. Mar. Biotechnol..

[88] Di Lena G., Casini I., Lucarini M., Lombardi-Boccia G. (2019). Carotenoid profiling of five microalgae species from large-scale production. Food Res. Int..

[89] Pisal D.S., Lele S. (2005). Carotenoid production from microalga, dunaliella salina. Indian J. Biotechnol..

[90] Ghaderpour S., Ahmadifard N., Agh N., Vahabzadeh Z., Estevez A. (2021). Short-term enrichment of microalgae with inorganic selenium and zinc and their effects on the mineral composition of microalgae and marine rotifer brachionus plicatilis. Aquac. Nutr..

[91] Masojídek J., Ranglová K., Lakatos G.E., Silva Benavides A.M., Torzillo G. (2021). Variables governing photosynthesis and growth in microalgae mass cultures. Processes.

[92] Geider R.J., La Roche J. (1994). The role of iron in phytoplankton photosynthesis, and the potential for iron-limitation of primary productivity in the sea. Photosynth. Res..

[93] Black J.R., Yin Q.-Z., Casey W.H. (2006). An experimental study of magnesium-isotope fractionation in chlorophyll-a photosynthesis. Geochim. Cosmochim. Acta.

[94] Vannucci L., Fossi C., Quattrini S., Guasti L., Pampaloni B., Gronchi G., Giusti F., Romagnoli C., Cianferotti L., Marcucci G. (2018). Calcium intake in bone health: A focus on calcium-rich mineral waters. Nutrients.

[95] Hodges J., Cao S., Cladis D., Weaver C. (2019). Lactose intolerance and bone health: The challenge of ensuring adequate calcium intake. Nutrients.

[96] Elmadfa P.D.I., Muskat P.D.E., Fritzsche D., Meyer A.L. (2021). Die Große gu Nährwert-Kalorien-Tabelle.

[97] Volpe S.L. (2013). Magnesium in disease prevention and overall health. Adv. Nutr..

[98] Sebastiani G., Herranz Barbero A., Borrás-Novell C., Alsina Casanova M., Aldecoa-Bilbao V., Andreu-Fernández V., Pascual Tutusaus M., Ferrero Martínez S., Gómez Roig M.D., García-Algar O. (2019). The effects of vegetarian and vegan diet during pregnancy on the health of mothers and offspring. Nutrients.

[99] Rayman M.P. (2012). Selenium and human health. Lancet.

[100] Kieliszek M. (2019). Selenium–fascinating microelement, properties and sources in food. Molecules.

[101] Lin P.H., Sermersheim M., Li H., Lee P.H.U., Steinberg S.M., Ma J. (2017). Zinc in wound healing modulation. Nutrients.

[102] Skrajnowska D., Bobrowska-Korczak B. (2019). Role of zinc in immune system and anti-cancer defense mechanisms. Nutrients.

[103] Fischer P.D.M., Glomb P.D.M.A. (2015). Moderne Lebensmittelchemie.

[104] Tripathy A., More R., Gupta S., Samuel J., Singh J., Prasad R. (2021). Present and future prospect of algae: A potential candidate for sustainable pollution mitigation. Open Biotechnol. J..

[105] Tchounwou P.B., Yedjou C.G., Patlolla A.K., Sutton D.J. (2012). Heavy metal toxicity and the environment. Exp. Suppl..

[106] Ratnaike R.N. (2003). Acute and chronic arsenic toxicity. Postgrad. Med. J..

[107] Fabregas J., Herrero C. (1986). Marine microalgae as a potential source of minerals in fish diets. Aquaculture.

[108] Pradhan B., Bhuyan P.P., Nayak R., Patra S., Behera C., Ki J.-S., Ragusa A., Lukatkin A.S., Jena M. (2022). Microalgal phycoremediation: A glimpse into a sustainable environment. Toxics.

[109] Spain O., Plöhn M., Funk C.J.P.P. (2021). The cell wall of green microalgae and its role in heavy metal removal. Physiol. Plant..

[110] Matissek R., Steiner G., Fischer M. (2010). Lebensmittelanalytik.

[111] Lee S.C., Prosky L., Vries J.W.D. (2020). Determination of total, soluble, and insoluble dietary fiber in foods—Enzymatic-gravimetric method, mes-tris buffer: Collaborative study. J. AOAC Int..

[112] Matyash V., Liebisch G., Kurzchalia T.V., Shevchenko A., Schwudke D. (2008). Lipid extraction by methyl-tert-butyl ether for high-throughput lipidomics. J. Lipid Res..

[113] Jeffrey S.W., Humphrey G.F. (1975). New spectrophotometric equations for determining chlorophylls a, b, c1 and c2 in higher plants, algae and natural phytoplankton. Biochem. Physiol. Pflanz..

[114] Jaspars E. (2006). Pigmentation of tobacco crown-gall tissues cultured in vitro in dependence of the composition of the medium. Physiol. Plant..

[115] Rowan K.S., Press C.U. (1989). Photosynthetic Pigments of Algae.

